# VizChemoton: Visualization of Quantum Chemical Reaction Networks and Their Cheminformatics Features

**DOI:** 10.1021/acs.jcim.6c02689

**Published:** 2026-09-15

**Authors:** Enric Petrus, Diego Garay-Ruiz, Thomas Weymuth, Markus Reiher, Thomas B. Hofstetter

**Affiliations:** † 28499 Eawag, Swiss Federal Institute of Aquatic Science and Technology, 8600 Dübendorf, Switzerland; ‡ 69583 Instituto de Micro y Nanotecnologıa, IMN-CNM, CSIC (CEI UAM+CSIC), Isaac Newton, 8, Tres Cantos, Madrid E-28760, Spain; § Department of Chemistry and Applied Biosciences, 31064 ETH Zürich, Vladimir-Prelog-Weg 2, 8093 Zürich, Switzerland; ∥ Institute of Biogeochemistry and Pollutant Dynamics (IBP), 27219 ETH Zürich, 8092 Zürich, Switzerland

## Abstract

Automated explorations of chemical reaction networks (CRNs) guided by quantum chemistry (QC) calculations are undergoing rapid development that enables numerous applications. However, CRNs are accessible primarily through specialized quantum chemistry software without interface for cheminformatic exploitation of the generated data. Here, we present VizChemoton, an open-source module within the reaction exploration framework *Software for Chemical Interaction Networks* (SCINE). VizChemoton enables visualization of CRNs and serves as an interface for cheminformatics applications based on conversion of molecular structures that accounts for radicals, zwitterions, and molecular complexes into string-based representations via RDKit mol objects. The conversion performance exceeded 90% of QC-generated compounds based on the evaluation of four CRNs constructed with density functional theory and extended tight-binding methods. We find that compounds absent from public structural databases correspond primarily to molecular complexes and unstable reaction intermediates. All essential QC and associated cheminformatics data are distributed in interoperable CSV and JSON formats to support downstream machine learning applications whereas reaction networks are readily visualized through a browser-based standalone HTML interface.

## Graphical abstract

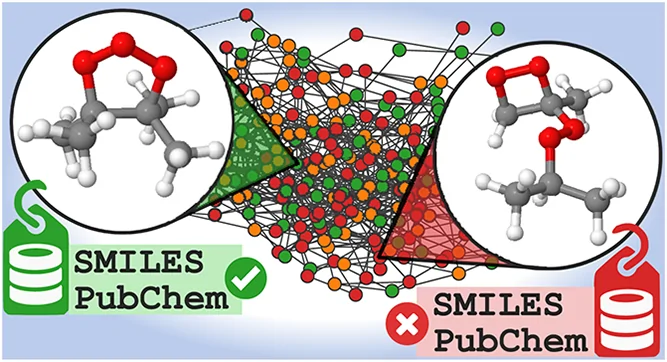

## Introduction

Chemical reaction networks (CRNs) are key to modeling and predicting chemical reactivity.^1,2^ Yet, recent advances rapidly transform the procedures how such networks are generated. Fast quantum chemistry (QC)-based methods, such as extended tight-binding approaches (xTB)^3,4^ and machine learning interatomic potentials (MLIPs),^5–7^ have reduced the computational cost relative to Density Functional Theory (DFT) and Wave Function Theory (WFT) calculations significantly, thereby enabling the construction of substantially larger reaction networks. Moreover, the development of autonomous frameworks facilitates the automated construction of CRNs by means of molecular dynamics simulations^8–12^ and automated scans of the potential energy surface.^13–15^ These advances have enabled CRN explorations in various fields, including materials discovery for Li-ion batteries,^16^ biomass pyrolysis,^17^ heterogeneous carbon dioxide electroreduction,^18^ and oxidative water treatment,^19^ with numerous applications to come.

Because automated CRN explorations inherently generate large amounts of data, they are frequently accompanied by specialized graphical user interfaces (GUIs) that facilitate the visualization of QC results through online web services,^20,21^ and haptic devices.^22^ The extensive chemical datasets produced by CRNs are also highly valuable for cheminformatics-based investigations of the relationships between two-dimensional (2D) molecular structures and chemical properties.^23,24^ In this context, encoding molecular structures as string-based representations, such as the Simplified Molecular Input Line Entry Specification (SMILES),^25^ SMILES Arbitrary Target Specification (SMARTS), and the International Chemical Identifier (InChI),^26^ is fundamental to the development of predictive models. For example, molecular representations used in Quantitative Structure–Activity Relationship (QSAR) models are essential for predicting chemical properties and the behavior of complex systems in cases where QC calculations may be inaccessible, particularly in drug design and medicinal chemistry.^27^ Such representations have also been extensively integrated with machine learning (ML) techniques.^28–32^

Despite these advances, tools that exploit QC-generated information through interfaces for cheminformatics applications and straightforward CRN visualization remain scarce.^33^ Achieving a balance between a user-friendly GUI that minimizes technical demands and provides application-specific information (e.g., displaying selected regions of CRNs and targeted chemical properties) remains highly desirable, yet challenging. Although most QC-related GUIs rely on molecular representations to enable interaction with CRNs, the conversion of QC-generated molecular structures into string-based formats is subject to inherent limitations as no information beyond bond connectivity and atom types is considered.^34^ Consequently, certain compounds cannot be adequately represented. SMILES, for example, cannot encode spin multiplicities or fully describe molecular adducts.^35^ The development of frameworks capable of converting large CRNs into string representations suitable not only for GUIs but also for seamless integration into ML workflows thus remains an open challenge.^36,37^

Here we present VizChemoton, a software tool designed to visualize QC-generated reaction networks together with their associated cheminformatics data in standalone files. VizChemoton lowers the technical barrier to accessing CRN data outside QC-specific GUIs and facilitates the transformation of CRNs into interoperable datasets suitable for subsequent ML applications. Our first objective was to implement methods for converting compounds within CRNs into string-based molecular representations through RDKit mol objects. To evaluate the performance of these structure-to-string conversion methods, we considered two medium-sized DFT-based CRNs and two large-scale xTB-based CRNs, all generated using Chemoton,^15,38^ the reaction exploration module of the open-source Software for Chemical Interaction Networks (SCINE).^39^ Second, we demonstrate the utility of RDKit mol objects in the context of automated reaction network exploration through two cheminformatics applications: the analysis of compound chemical space across different CRNs and public chemical structure databases, and the evaluation of selected molecular properties. Third, we improve the interoperability of SCINE CRN results by providing two plain-text output formats (CSV and JSON), thereby enabling seamless integration into ML workflows. Finally, we combine QC and cheminformatics results within a standalone HTML-based GUI, providing a lightweight alternative to the main Heron interface.^22^

## Reaction Exploration Details

The CRNs evaluated in this work were constructed using Chemoton^15,38^ version 3.1.0,^40^ the open-source reaction exploration module in SCINE.^39^ Reaction network data was stored in a MongoDB, and accessed using SCINE Database version 1.3.0.^41^ Details on the quantum chemistry methods employed and their computational cost during the reaction explorations are available in the Supporting Information (Section S2). These CRNs were originally set up to study the oxidative water treatment process in which either ethene or tetramethylethene reacted with ozone and are referred to here as CRN-E and CRN-T, respectively.^19^ Table 1 summarizes features of the CRNs based on calculation with DFT and xTB for CRN-E and CRN-T, respectively. The rationale for using these four CRNs as basis for testing their conversion to molecular representations used in cheminformatics was as follows. First, these CRNs represent extensive evaluations of olefin ozonation chemistry that successfully reproduced experimental observations.^19^ Second, the large CRNs generated with xTB ensure comprehensive study of the numerous reaction products and thus represent a suitable case to test cheminformatics and visualization workflows implemented in VizChemoton.

**Table 1:** Quantum Chemistry and Cheminformatics Features of the Four Studied Chemical Reaction Network Explorations for Ozone and Ethene (CRN-E) and Ozone and Tetramethylethene (CRN-T) Using DFT and xTB, Respectively. Computational Cost Expressed as Serial Computing Time and Wall Clock Time. Size of the CRNs Described by the Number of Reactions, Compounds and Flasks (Non-Bonded Complexes). Performance of Three Methods for Converting Compounds to RDKit Mol Objects. Compound Chemical Space Coverage between DFT and xTB, and in Three Public Structural Databases

	CRN-E		CRN-T	
	DFT	xTB	DFT	xTB
**Computational cost:**				
Serial computing time (days)	28505	2784	11653	1436
Wall clock time (days)^a^	114.0	2.8	46.6	1.4
**Size of the chemical reaction networks:**				
Number of reactions	6159	78950	477	123794
Number of compounds and flasks (100%)	1743	40676	415	74861
**Conversion to SMILES/RDKit mol objects:** ^b^				
xyz2mol	1227 (70%)	25084 (62%)	394 (94%)	47739 (64%)
SCINE-BOs	1030 (60%)	28454 (70%)	275 (66%)	55883 (74%)
Hybrid	1459 (84%)	36860 (91%)	410 (99%)	68086 (91%)
**Compound chemical space coverage:** ^c^				
Total number of InChIKeys for compounds and flasks^d^	880	29525	363	45757
Common InChIKeys between DFT and xTB	443 (50%)		220 (61%)	
PubChem	378 (42%)	1567 (5%)	114 (31%)	6260 (14%)
ChEBI	81 (9%)	162 (0.5%)	24 (7%)	457 (1%)
ChEMBL	47 (5%)	105 (0.3%)	19 (5%)	301 (0.6%)

a DFT and xTB-CRN calculations were performed on 250 and 1000 cores, respectively, using AMD EPYC 7742 CPUs.

b Percentages refer to the total number of unique compounds and flasks defined by Molassembler.

c Percentages refer to the total number of unique InChIKeys obtained from the RDKit mol objects generated with the Hybrid method.

d If flasks are not accounted for, percentages depicted below increase an average of 10% to 15%.

## Code Design

VizChemoton starts by extracting the reaction network data generated by Chemoton and stored in the MongoDB, via the SCINE Database. Then it proceeds with data processing, which is divided into two parts: (i) conversion of SCINE compounds to cheminformatics data such as RDKit mol objects and matches in public structural databases and (ii) conversion of the CRNs to an interactive HTML visualization dashboard. VizChemoton generates three standalone and interoperable output files: *compounds.json*, *reactions.csv* and *network.html*. The source code is delivered as VizChemoton version 2.1 (version 1.0 corresponds to a prototype without an associated article^42^) and is freely accessible as part of the SCINE suite of modules (see more details in Figures S1–S3).

### Cheminformatics

We have evaluated the performance of converting QC structural information, calculated during reaction exploration, into two-dimensional molecular graphs, processed as RDKit mol objects, thus unlocking access to a wide variety of cheminformatic tools.^43,44^ Our benchmark discussed in Section S3 showed that a combination of QC bond orders and rule-based prediction of bond orders,^45^ denoted as “Hybrid”, provided a robust and consistent conversion to RDKit mol objects. Figure 1 summarizes the Hybrid method algorithm for deducing the InChIKey and SMILES of the new structures found in the reaction exploration, interfacing the already existing SCINE (green boxes) and RDKit (blue boxes) libraries. SCINE-BOs are converted to MOL format using SCINE Utilities, which rounds the bond orders to the nearest integer. The MOL format is imported by the RDKit built-in function using the recommended default parameters, which ensure that the chemical structure undergoes sanitization (i.e., checks if a molecule is chemically plausible, and defines aromaticity, valence and hybridization), removes explicit hydrogens and converts them into an internal property (thereby reducing the molecular graph size), and performs strict parsing, ensuring no deviation from the Chemical Table (CTAB)^46^ format is tolerated. If conversion from MOL for RDKit mol object fails and the compound is a singlet, we rely on the rule-based prediction of bond orders developed by Kim and Kim^45^ and implemented in RDKit as xyz2mol^47^ (more details in Section S3).

**Figure 1:**
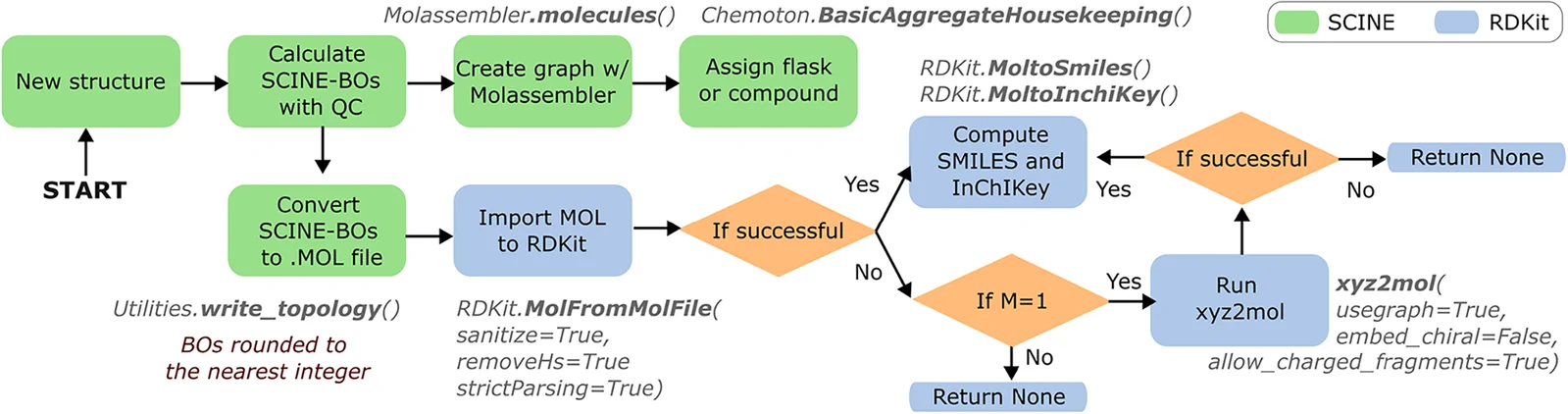
Algorithmic description of the Hybrid method used in this work to convert chemical structures −obtained during the reaction exploration with SCINE– to SMILES and InChIKeys via RDKit mol objects.

The performance of the Hybrid method is shown in Table 1. These data illustrate the increased share of converted compounds by 5% (CRN-T-DFT) to 21% (CRN-E-xTB) of the number of structures and flasks (i.e., non-bonded complexes in SCINE notation^15^). Figure S9 shows the breakdown of the number of non-converted compounds for CRN. This increase in compound conversion is based on the fact that the Hybrid method correctly transforms both singlet zwitterionic compounds and high-multiplicity compounds into RDKit mol objects. For instance, the Hybrid method successfully converted doublet compounds such as the methyl radical, [H_3_ C·], hydroxyl radical, [HO·], and methoxy radical, [CH_3_O·], because SCINE-BOs were derived from quantum chemical calculations that explicitly account for the spin state. Moreover, the Hybrid method successfully converted zwitterions (Figure S6) because, when SCINE-BOs could not be converted into an RDKit mol object, it relied on xyz2mol. The latter assigns formal charges to atoms in charged fragments that do not satisfy standard neutral valences. We further evaluated the failed singlet cases for xyz2mol (Figure S7) and the failed non-singlet cases for SCINE-BOs (Figure S8). In both data sets, more than 60% of the failed cases correspond to flasks. This finding is consistent with string molecular representations available in RDKit mol objects (e.g., SMILES), where non-covalent interactions are not considered as part of the chemical graph.^25^

#### Integrations for Compound Chemical Space Comparison

VizChemoton contains two specific integrations for comparing the compound chemical spaces between (i) CRNs calculated with different electronic methods, and (ii) with public structural databases. To contrast the compound chemical space of two CRNs calculated with different electronic methods (e.g., DFT and xTB), we could not rely on the ID serialization number created by Molassembler because structures optimized with different electronic methods render different IDs. Furthermore, we could not use the SMILES notation available in the RDKit mol objects because it is a noncanonical compound representation,^34^ and therefore it is not suitable for a direct compound comparison. To bypass the ambiguity inherent to SMILES, we employed InChIKey identifiers, a hashed, more compact version of InChI strings. InChIKey is intended for searching the internet and indexing ofdatabases. The collision risk, that is, an InChIKey pointing to two or more InChI, is minimal and might occur only for isomers of very large molecules.^48^ Moreover, we integrated the Python Application Programming Interfaces (APIs) of PubChem, ChEMBL, and ChEBI as part of the VizChemoton workflow in order to compare the compounds in CRNs with the vast resources of public databases. PubChem contains comprehensive information on chemicals and their biological activities,^49,50^ ChEMBL lists drug-like bioactive compounds,^51^ and ChEBI covers chemical entities of general biological interest.^52^

### Visualization

The GUI rendered by VizChemoton enables simple queries including SMILES-, InChIKey- and ID-based compound searches by representing QC and cheminformatics data in a standalone HTML file. This GUI complements the existing SCINE GUI Heron, which allows exhaustive access and visualization of all the QC results generated during the reaction exploration. However, Heron requires continuous connection to the MongoDB instance where the reaction network is stored, and thus prohibits the standalone and interoperable use of the CRN results.

The GUI layout consists of an HTML file created using the open-source library amk-tools,^53^ so that the CRN data can be accessed in an interactive manner using an internet browser (internet only needed for automatically loading the JavaScript modules). The HTML dashboard includes a search feature, allowing the user to rapidly check whether a compound of interest was formed during the CRN exploration. We also implemented a workflow to map qualitative (Figure S17) and quantitative (Figure S15) cheminformatics results on top of the nodes in the HTML visualization, as well as visualizing the most likely reaction pathways between a pair of predefined source and target compounds (Figure S18). Another important point concerns the size of the network as it plays a critical role in the distribution of nodes and edges on the HTML canvas. For small-to-medium sized networks such as DFT-CRNs, we rely on the Kamada Kawai^54^ and Fruchterman-Reingold^55^ algorithms as implemented in NetworkX.^56^ However, for large networks such as our xTB-CRNs, these algorithms become impractical due to the use of large amounts of memory. Therefore, we implemented a k-means clustering^57^ to set similar molecular structures closer on the canvas using basic structural descriptors derived from the Cartesian coordinates of the molecular structures (Figure S15). A detailed description of the features available in the HTML file can be found in Section S5.

### Interoperability

VizChemoton manages the transformation of key QC and cheminformatics results, including network topology, to plain text format files that can be opened by third-party tools, thereby ensuring interoperability. In fact, simulations performed with SCINE are findable and accessible in Zenodo^58^ and ioChem-BD,^59^ and the results are reusable, provided that the same versions of SCINE modules are used. Hence, the interoperability aspect complements the existing features of CRN construction to fulfill the FAIR principles.^60^

Information in VizChemoton is organized in three independent files, describing *compounds*, *reactions* and the *network*. The *compounds.json* file contains explicit structural and SCINE-related information (Cartesian coordinates, MongoDB ID), QC relevant data (electronic energy, spin state, basis set), and cheminformatics data (SMILES, InChIKey, public database IDs). This file also has implicit information about the reaction network, defining nodes for reaction complexes (reactants or products) that might contain several compounds. For unimolecular reactions, one node will correspond to one compound, but for bi- and termolecular reactions, these nodes will be composed of two and three compounds, respectively. The second file, *reactions.csv*, contains explicit reaction information, consisting of a simple enumeration of which nodes (matching the definitions in the *compounds* file) are involved in which reactions. See Section S1 for more details on the format of the JSON and CSV files. The third file corresponding to the GUI is implemented as an HTML file that can be readily rendered from the JSON and CSV outputs (no connection to MongoDB required), enabling rapid customization of the properties to display.

## Application Examples

### Analyses of Compound Chemical Spaces

The comparison of DFT- and xTB-generated reaction networks is of particular interest for determining efficient strategies for CRN construction through the combination of fast and accurate methods. Here, we addressed this question by analyzing compound chemical spaces obtained with xTB and DFT methods. We computed the overlap between these two networks using the InChIKeys from the RDKit mol objects, which enabled an unambiguous compound comparison. Table 1 shows that CRN-E-DFT shares 443 InChIKeys with CRN-E-xTB (50% of DFT-InChIKeys) and CRN-T-DFT shares 220 InChIKeys with CRN-T-xTB (61%), indicating that xTB finds approximately half of the DFT compound chemical space. Common compounds found with both methods correspond to typical ozonation byproducts such as formaldehyde (C=O), ozonide (CC1OOO1) and acetone (CC­(C)=O). When flasks are excluded from this comparison, the overlap in chemical space between the DFT and xTB networks increases to 66% and 64% for CRN-E and CRN-T, respectively. Furthermore, we created four barcode-like representations sorted by the number of atoms in Figure S10 to visualize the common vs. overlooked compounds in DFT and xTB reaction networks. This evaluation showed that xTB did not disregard specific regions of the DFT networks within the atom stoichiometry boundary conditions defined at the start of the reaction exploration. xTB-based CRN evaluations thus cover approximately two thirds of the DFT chemical space in one tenth of the DFT simulation time. As postulated in our earlier work,^19^ this observation confirms the sequential implementation of xTB (for CRN exploration) and DFT (for structure refinement) as strategy for efficient construction of CRNs for ozonation chemistry.

We also examined the question of experimental coverage vs. the discovery of novel compounds in the compound chemical spaces of the SCINE CRNs. We posit that large public structural databases could, in principle, cover large shares of compounds in SCINE CRNs once flasks and reactive intermediates (such as organic peroxides and ozonides) are excluded. To that end, we have compared all compounds that could be converted to RDKit mol objects for the four CRNs, to three deliberately chosen structural databases with relevance to the chemistry of oxygenated organic compounds, namely PubChem, ChEMBL, and ChEBI. Table 1 shows the number of InChIKeys found in the three public databases for the four CRNs. PubChem was the database with the highest number of matches even though these shares were relatively low and ranged from 5% to 42% of InChIKeys in the four CRNs. Matches with ChEMBL and ChEBI databases were substantially lower (i.e., from 0.3% to 9%). This outcome is consistent with the fact that PubChem is significantly larger (≈ 111 million compounds) than ChEMBL (≈ 1 million) and ChEBI (≈ 195000). Because xTB-CRNs are larger than DFT-CRNs, the absolute number of matches is also higher while their shares are smaller. For example, CRN-T-xTB contains 6260 matches in PubChem out of a total of 45757 (14%) as compared to 114 matches (31%) for CRN-T-DFT.

We analyzed the origin of this mismatch using the SMILES arbitrary target specification (SMARTS), a common approach to automatically search for substructures (i.e., chemical patterns) in string-based molecular representations.^61^ Specifically, we selected four SMARTS that we hypothesized to be infrequent in experimental databases. These included (i) flasks, which correspond to non-bonded complexes, (ii) organic peroxides, which tend to hydrolyze in water,^62^ (iii) ozonides, which undergo heterolytic cleavages,^63^ and (iv) other peroxides, which are common short-lived intermediates that form during ozonation reactions.^64,65^ Figure 2 shows that most of the mismatches (45% to 60%) correspond to entries associated with flasks denoted in the general form A.B regardless of the CRN. These adducts are essential from the QC point of view but cannot be included in the chosen databases for individual molecules. In addition, the various peroxides and ozonides make up a significant fraction of the InChIKeys not found in PubChem (Figure 2). Most of them are organic peroxides of SMILES general form [CX4]-O-[O;D1], in agreement with well-known tendencies of ozonation reactions to form hydrogen peroxide and hydroperoxo compounds and ozonides.^62,63,66^ The O–O and O–O–O bond patterns are essential in the context of ozonation reactions, but these compounds rapidly decompose to hydrogen peroxide and/or molecular oxygen.^64,65^ Consequently, these compounds are less likely to be found in experimental databases such as PubChem.^67^ Taken together, flasks, (organo)­peroxides, and ozonides make up for 83% to 97% and thus a substantial share of the InChIKey-encoded compounds of the CRNs that were not found in PubChem (see “sum” in Figure 2).

**Figure 2:**
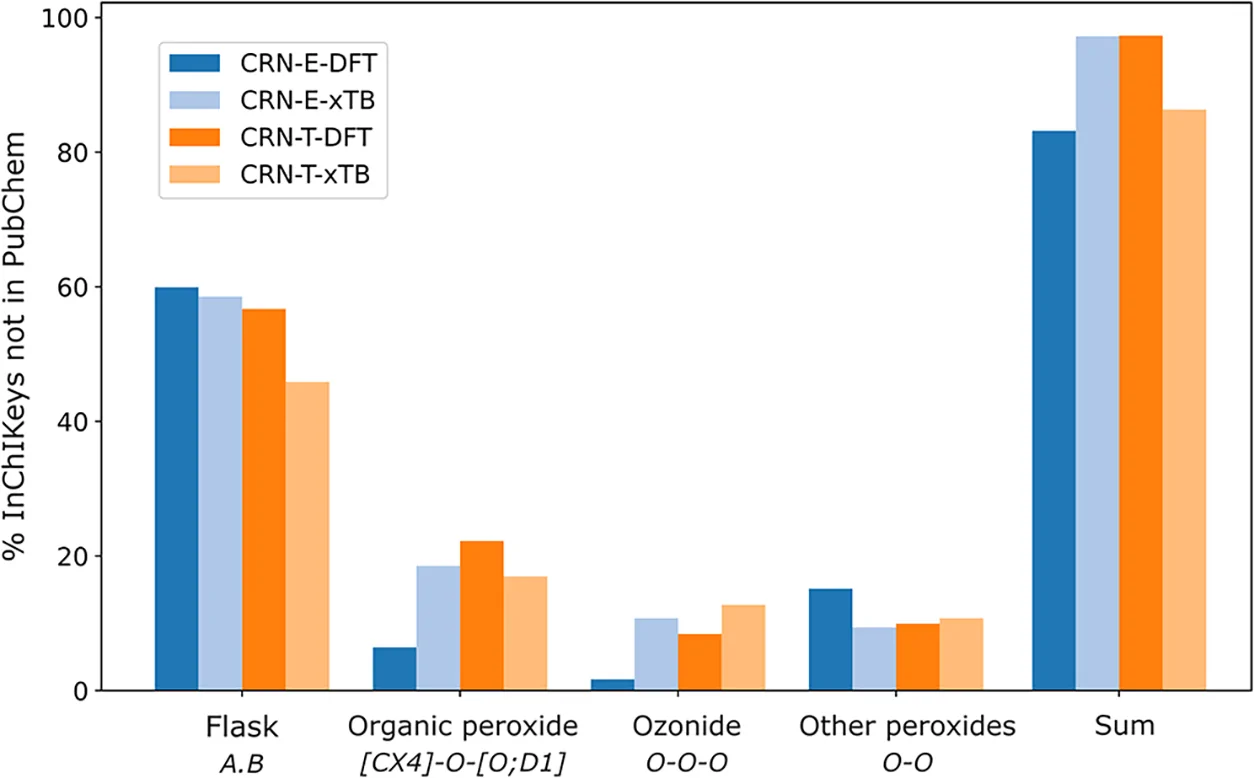
Percentages of InChIKeys that were not found in PubChem regarding the four CRNs considered in this study. Not found InChIKeys are classified according to three SMARTS patterns (organic peroxide, ozonide and other peroxides) and on whether they are a flask (A.B). A sum of the not found InChIKeys percentages is provided on the right.

Structures predicted by SCINE but not indexed in publicly accessible databases can also guide the identification of unknowns, for example, by enriching structural databases for mass spectrometry-based analysis of unknown metabolites and transformation products. To that end, we tested a downstream ML application in which the SMILES representations of CRN-T-xTB structures were introduced as a custom database in Sirius^68^ to elucidate the structure of theunannotated MS^2^ spectrum of 2-pentanone (see Section S4.4 and Figure S13). The structure predicted by Sirius using the CRN-T-xTB-generated database showed higher similarity to 2-pentanone than the structure predicted without the additional database entries. This seamless integration was enabled by the interoperable JSON output file provided by VizChemoton, which can be readily converted into other formats, such as the TSV format required by Sirius.

### Chemical Property Descriptors

We explored selected chemical property descriptors readily accessible from the RDKit mol objects generated with the Hybrid method for the two xTB reaction networks. Figure S12 shows histograms for logP, the octanol-water partition coefficient describing a compound’s distribution in biota-like systems,^69^ QED, the quantitative estimation of drug-likeness, which covers multiple molecular properties of relevance in drug discovery and biochemistry,^30^ and SPS, the spacial score, which characterizes molecular complexity.^70^ The distribution of logP-values illustrates the higher tendency of compounds in CRN-T to partition into an organic solvent as compared to compounds in CRN-E which show a preference for associating with water. This outcome is consistent with the carbon–oxygen atom stoichiometry in tetramethylethene (CRN-T) vs. ethene (CRN-E) as organic reactants. Indeed, compounds in CRN-T contain more carbon atoms, a higher carbon–oxygen ratio, and are larger than compounds in CRN-E (see Figure S10). All of these properties favor larger logP-values.^69^ The same trend is observed for the QED distribution, although this drug similarity descriptor is a composite measure with a more nuanced measure for the criteria to increase the bioavailability of a compound. Finally, the distribution of compounds in SPS scores of CRN-E represents a subset of the one for CRN-T. Despite its simplicity, this preliminary analysis highlights the potential of VizChemoton as a valuable tool to make information from QC reaction networks accessible to cheminformatics applications.

### Standalone VizChemoton GUI

We integrated results from QC CRNs and cheminformatic applications in VizChemoton’s standalone GUI. With this GUI, it is possible to visualize entire and/or specific parts of the reaction network, access to precalculated cheminformatic properties, and search for specific compounds of the network using the SMILES or InChIKey notation. Compounds that could not be converted to RDKit mol objects are indexed as None. The full description of the visualization features is given in Section S5. Figure 3 shows an example featuring CRN-T-DFT centered on the ozonide compound, with SMILES CC1­(C)­OOOC1­(C)­C. It also shows its two closest reaction layers, where a reaction layer is defined as the set of reactions connected to a compound. Layer 1 comprises reactions involving CC1­(C)­OOOC1­(C)­C whereas layer 2 comprises reactions involving compounds connected to CC1­(C)­OOOC1­(C)­C. The SMILES search feature in the HTML is particularly useful for simple line-notation queries which can inform users whether a transformation product was found, and if so, through which reaction(s).^71,72^

**Figure 3:**
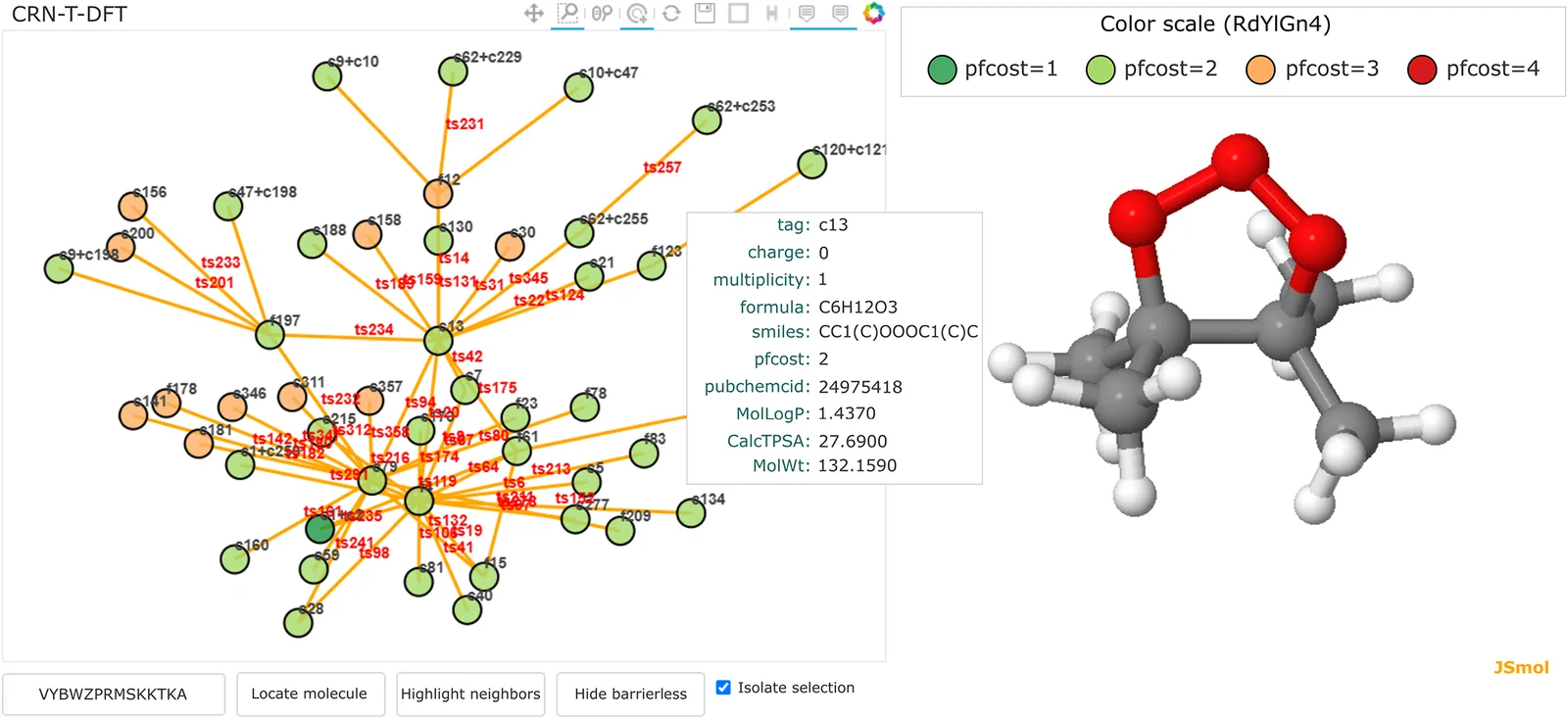
VizChemoton HTML dashboard for CRN-T-DFT centered on the ozonide and with the two adjacent reaction layers. Ball-and-sticks representation of the ozonide on the right. Color nodes indicate compound availability derived from the Pathfinder cost. Low compound costs (in green) indicate high availability, whereas high compound costs (in red) indicate low availability. Pathfinder compound cost scores (pfcost) are used to illustrate order-of-magnitude differences in compound costs and thus availability.

The fact that a compound is found in the network, however, does not *per se* imply thermodynamic and/or kinetic relevance, as VizChemoton depicts the complete CRN that has been computed. To estimate chemical relevance, we calculated Pathfinder compound costs for all nodes in the network, a measure of compound availability derived from reaction barrier heights and a set of initial costs.^73^ In Figure 3, the initial costs were assigned to reflect an initial 100-fold excess of ozone and water relative to tetramethylethene.^19^ Because compound costs typically span multiple orders of magnitude, we plot their decimal logarithms as relative pfcost labels to facilitate visualization. Greener colors indicate lower compound costs and, therefore, higher availability, whereas redder colors indicate higher compound costs and, consequently, lower availability. Furthermore, compound costs can be leveraged to identify the most likely reaction pathways between a source and a target compound through the reaction profile feature (Figures S14 and S18).

## Conclusion

VizChemoton is a new light-weight GUI for the SCINE framework that enables simple access to QC-generated CRNs and provides an interface for cheminformatic exploitation of the associated QC-data. We implemented a Hybrid method combining QC-derived (SCINE) bond orders calculated on-the-fly during CRN exploration with rule-based (xyz2mol) bond orders to account for unpaired spin compounds and zwitterions in the generation of string molecular representations (SMILES, SMARTS, InChIKeys) with an average conversion rate of 91% of the computed compounds. We compared InChIKeys in the CRNs against three public structural databases —PubChem, ChEMBL, and ChEBI— using their respective APIs. Missing compounds in PubChem, were traced back to non-bonded complexes and reactive intermediates (here peroxides and ozonides) which are typically not reported in such compound repositories. Our study illustrates how QC-generated data can be leveraged through tools such as VizChemoton that allow a variety of applications, including the augmentation of structural databases and ML workflows for unknown compound identification.

## Supplementary Material



## Data Availability

The source code of VizChemoton version 2.1.0 is available at https://github.com/qcscine/vizchemoton. All data related to this work will be accessible at 10.25678/000H1V.

## Notes

Description of the VizChemoton codebase; computational details; conversion of compounds into SMILES/RDKit mol objects, comparison of DFT and xTB compound chemical spaces; identification of compound matches in public structural databases; and visualization features of the HTML GUI (PDF)
