# Supplementary material for: VizChemoton: Visualization of Quantum Chemical Reaction Networks and Their Cheminformatics Features
Source: J Chem Inf Model. 2026 Sep 15;66(18):11596–604. doi: 10.1021/acs.jcim.6c02689 (PMC13625967; doi:10.1021/acs.jcim.6c02689)
Supplement: Supplementary file 1 [file ci6c02689_si_001.pdf]

Supporting Information

VizChemoton: Visualization of Quantum Chemical Reaction Networks and Their Cheminformatics Features

Enric Petrus,[*,†] Diego Garay-Ruiz,[‡] Thomas Weymuth,[¶] Markus Reiher,[¶] and Thomas B. Hofstetter[*,†,§]

†*Eawag, Swiss Federal Institute of Aquatic Science and Technology, 8600 Dübendorf, Switzerland*

‡*Instituto de Micro y Nanotecnología, IMN-CNM, CSIC (CEI UAM+CSIC), Isaac Newton, 8, Tres Cantos, Madrid, E-28760, Spain*

¶*Department of Chemistry and Applied Biosciences, ETH Zürich, Vladimir-Prelog-Weg 2, 8093 Zürich, Switzerland*

§*Institute of Biogeochemistry and Pollutant Dynamics (IBP), ETH Zürich, 8092 Zürich, Switzerland*

E-mail: enric.petrus@eawag.ch; thomas.hofstetter@eawag.ch

28 Pages, 18 Figures

Contents

S1 Code Design **3**
S1.1 Input file: YAML . . . 3
S1.2 Output files: JSON and CSV . . . 6

S2 Computational Details **8**

S3 Conversion of Compounds to SMILES/RDKit Mol Objects **9**
S3.1 RDKit mol object conversion . . . 9
S3.2 Singlet compounds: SCINE-BOs vs xyz2mol . . . 11
S3.3 Non-singlet compounds: SCINE-BOs limitations . . . 13
S3.4 Hybrid approach: SCINE-BOs and xyz2mol . . . 14

S4 Application Examples **17**
S4.1 DFT and xTB compound chemical spaces . . . 17
S4.2 Public structural databases . . . 18
S4.3 Chemical property descriptors . . . 19
S4.4 Machine learning . . . 19

S5 Visualization Features **20**
S5.1 General layout . . . 20
S5.2 Quantitative mapping . . . 22
S5.3 Large chemical reaction networks . . . 22
S5.4 Qualitative mapping . . . 24
S5.5 Reaction profiles . . . 25

References **27**

27

S1 Code Design

In this section, we provide an overview of the code design, as well as the input and output files handled by VizChemoton. Figure S1 depicts the code workflow in the context of the SCINE framework,[1] as well as the three standalone files (CSV, JSON and HTML) that it generates. The CSV and JSON files are described in section S1.2 and the HTML file is explained in section S5.

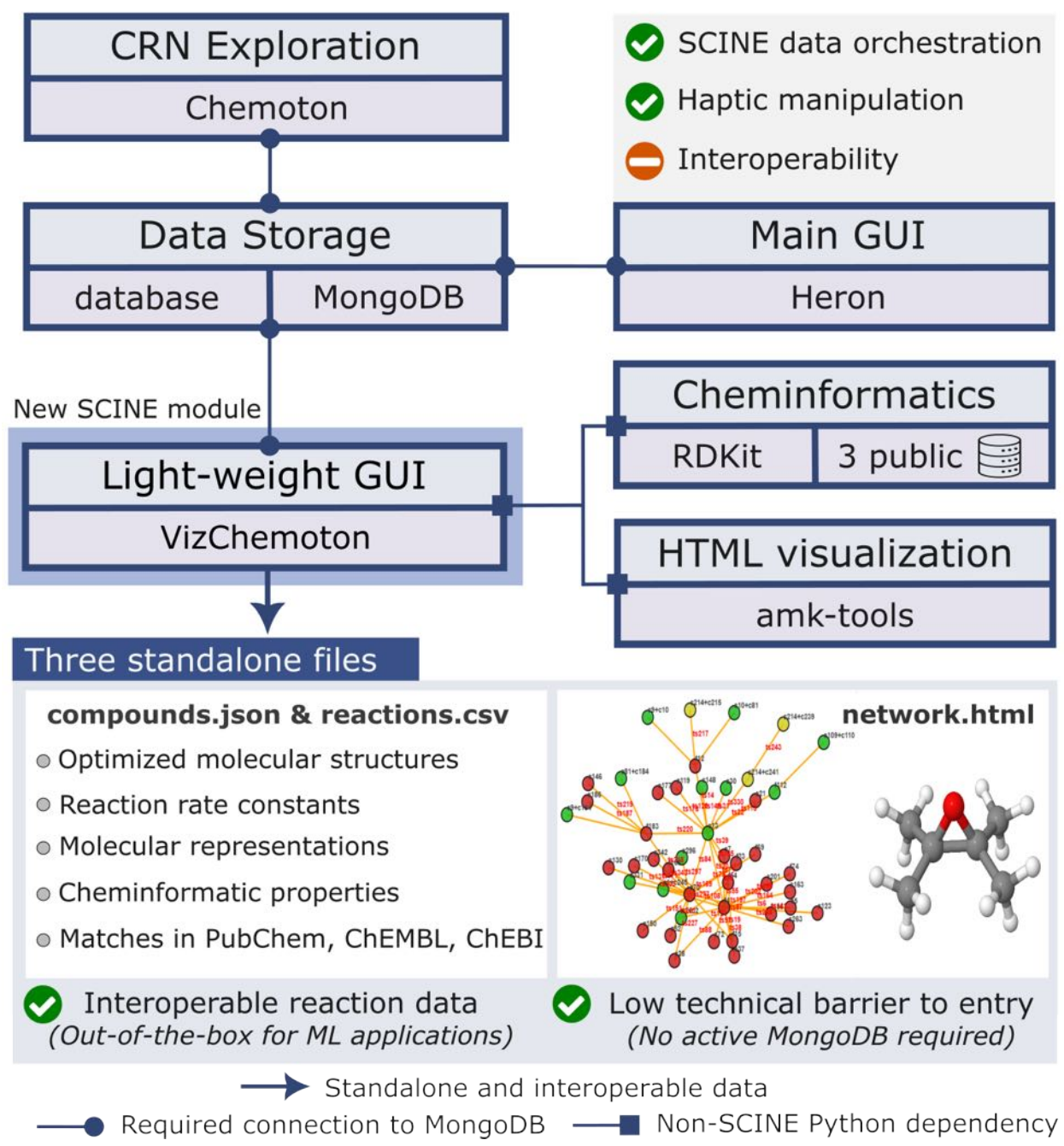

Figure S1: Overview of the module dependency of VizChemoton arranged according to their task classes. New module developed in this study is highlighted with a light blue frame. Circle-headed arrows indicate dependency on a MongoDB instance where the reaction network is stored, squared-headed arrows indicate external (non-SCINE) dependency, and normal arrows indicate standalone and interoperable data generated with VizChemoton.

S1.1 Input file: YAML

The input file of VizChemoton consists of a single YAML file with four sections: `scine`, `cheminfo`, `files` and `html` (Figure S3). The complete workflow of VizChemoton starts by extracting the chemical data in `scine`, generating the cheminformatics results in `cheminfo`, storing the results as JSON and CSV (optionally the network can also be saved in GRAPHML

```yaml
scine:
  active: True
  name: "ozone_tme_lcpbe"
  ip: "localhost"
  port: "27017"
  verbose: True
  method:
    method_family: "dft"
    method: "lc-pbe"
    basis_set: "def2-svp"
    program: "orca"
  pathfinder:
    mode_graph: "write"
    path_graph: "./vizchemoton/resources/pathfinder_graph_tme_dft.json"
    mode_costs: "write"
    path_costs: "./vizchemoton/resources/pathfinder_costs_tme_dft.json"
    init_costs: {"660d49de86b7e52a0c3bc06c": 100, # ozone
                 "660d4a1086b7e52a0c3bc070": 1,   # tme
                 "6649c98d86b7e5495e6e44ac": 100} # water

cheminfo:
  verbose: False
  rdkit:
      active: False
      method: "hybrid"
      props: ["MolLogP", "CalcTPSA", "MolWt"]
  database:
      pubchem: False
      chembl: False
      chebi: False

files:
  mode_reactions: "write"
  path_reactions: "./vizchemoton/resources/reactions_tme_dft.csv"
  mode_compounds: "write"
  path_compounds: "./vizchemoton/resources/compounds_tme_dft.json"
  path_graphml: "./vizchemoton/resources/network_tme_dft.graphml"

html:
  pathsearch:
    use_costs: True
    Npaths: 5
    max_length: 6
    source: "c1"
    target: "c48"
  dist_adduct: 3.0
  layout: "kamada_kawai"
  map_field: "pfcost"
  title: "Ozonation of Tetramethylethene with Chemoton"
  path_network: "./vizchemoton/resources/network_tme_dft.html"
```

Figure S2: Input YAML file of VizChemoton 2.1.

format) files in `files`, and rendering the HTML GUI in `html`. The modular design of the YAML file enables the user to speed up the workflow by either reading a previously generated pathfinder JSON file (which already indicates the lowest energy elementary steps for each reaction), or alternatively, render the HTML directly from the CSV and JSON files, without the requirement to use the `scine` and `cheminfo` sections. Figure S3 depicts the most important parameters for running VizChemoton, but the user can find more information about the YAML parameters in the code documentation.[2]

The first section is `scine`, where the user specifies if VizChemoton should connect to a MongoDB instance (`active`), and if so, the name of the database (`name`), the IP (`ip`) and port (`port`) of the server hosting the database. Because a reaction network can be calculated with multiple quantum chemistry methods, the user must specify which method was used, by means of the method type (`dft`), the specific method (`lc-pbe`), the basis set (`basis_set`) and the program (`program`). The name conventions are the same as in Chemoton. The next step is to calculate the reaction network graph (`path_graph`) with Pathfinder, which determines the elementary step of lowest-energy for every reaction in the network. It is also possible to calculate the compound costs (`path_costs`), that is, the availability of each compound in the network given some predefined initial concentrations (`init_costs`). Due to the increasing cost of calculating the network graph and compound costs for larger networks, it is possible to import previous runs (`mode_graph` and `mode_graph`).

The second section is `cheminfo`, where the user specifies if VizChemoton should calculate cheminformatic properties using RDKit (`active`). If so, the method to calculate the RDKit mol objects (`method`) and the desired properties (`props`) must be specified. The name conventions for the cheminformatic properties follow the exact function names depicted in the `Chem` library in RDKit. Furthermore, if RDKit mol objects were calculated, it is possible to query three public databases to determine which compounds in the reaction network are reported in PubChem (`pubchem`), ChEMBL (`chembl`) and ChEBI (`chebi`).

The third section is `files`, where the user specifies if VizChemoton should either write

or read (`mode_reactions` and `mode_compounds`) new or previously-generated pairs of output CSV and JSCON files (`path_reactions` and `path_compounds`). Notice that the read mode runs without an active connection to SCINE, which makes the resulting CSV and JSON files interoperable for other software. Furthermore, the reaction network graph can be stored in GRAPHML format so that it can be visualized with standard software such as Gephi.

The fourth and last section is `html`, where the user specifies the visualization features of the HTML GUI. The key parameters are `dist_adduct`, which defines the default distance (in Angstroms) between two compounds conforming an adduct (the larger the compounds, the higher this number should be), `layout`, which defines how nodes should be distributed in the canvas, `map_field`, which indicates which property should be colored map on top of the nodes, `title`, defining the title of the HTML, and `path_network` which indicates where should the HTML be stored. Furthermore, it is possible to calculate the reaction pathways, `pathsearch`, between a source and a target compound in the reaction network, specifying the use of compound costs, `use_costs`, the number of paths, `Npaths` and the maximum length of the network, `max_length`. Notice that the HTML can be generated by either going through the entire workflow running from `scine` to `html` (in `write` mode) or by processing the JSON and CSV output files (in `read` mode). This ensures that the user can easily and rapidly create different versions of the HTML network (including distinct reaction path searches) without having to connect to an active MongoDB database.

S1.2 Output files: JSON and CSV

VizChemoton converts the seven data collections in SCINE MongoDB to two interoperable text files that encode information about the CRN (Figure S3). To perform this data compression, we considered a graph where we grouped reactants and products into nodes, and reactions into edges. The first file corresponds to *compounds.json*, which contains information on all nodes in the graph (Figure S3A). Each node number has a defined number of keys which are sorted alphabetically. It contains the cheminformatics properties (SMILES,

A


```
  "9": {
    "CalcTPSA": 34.14,
    "MolLogP": 0.067,
    "MolWt": 31.998,
    "basis_set": "def2-svp",
    "charge": 0,
    "chebi": false,
    "chembl": false,
    "crn_id": "c9",
    "energy": -393213.0495035616,
    "inchikey": "MYMOFIZGZYHOMD-UHFFFAOYSA-N",
    "method": "lc-pbe",
    "mongodb_id": "6649c98d86b7e5495e6e4474",
    "multiplicity": 1,
    "pfcost": 2.387,
    "program": "orca any",
    "pubchem": false,
    "smiles": "O=O",
    "solvation": "none",
    "solvent": "none",
    "xyz": [["O",[-0.45567087460259315,-0.04297978523329241,-1.0104271139895828]],["O",[0.45567087460
259315,0.04297978523329241,1.010427113989583]]],
    "xyzdes": [2,2,1.1092547771297223,0.15831078050333758]
```


B


```
reactant, product, transition_state, rxn_id, es_id
3,4,None,6649c99486b7e549636e4424,661661120673b72785156f0b
4,5,6,6649c99486b7e549636e4426,6650cbae4640597cd479439c
4,7,8,6649c99486b7e549636e4428,66503c6c5029f9737d0b27fc
11,12,None,6649c99486b7e549636e442a,66169adf13beac7274797cbe
12,13,14,6649c99486b7e549636e442c,664b53ee9298f002d618e46f
15,18,None,6649c99486b7e549636e442e,6616b40ad3e5e66bba1dab4f
4,15,19,6649c99486b7e549636e4430,666e4f0edf2514688c6e95b0
4,13,20,6649c99486b7e549636e4432,664ee223ce2533010016a9d9
13,21,22,6649c99486b7e549636e4434,6617063a76ab0b7f366f65b8
23,26,None,6649c99486b7e549636e4436,66170713dd59ae096b23888b
3,23,27,6649c99486b7e549636e4438,66170713dd59ae096b23888c
3,28,29,6649c99486b7e549636e443a,6657f0398cd161106b207a45
13,30,31,6649c99486b7e549636e443c,66700c4804fb3a64a469c0e5
33,34,None,6649c99486b7e549636e443e,66173cc19afba012f5618067
35,38,None,6649c99486b7e549636e4440,66173cc19afba012f5618068
34,35,39,6649c99486b7e549636e4442,664d0ab8d578bf0c45663596
4,40,41,6649c99486b7e549636e4444,6653b9cece365e3ee94bbf0c
13,7,42,6649c99486b7e549636e4446,66178969da807e2ec23d27f5
44,45,None,6649c99486b7e549636e4448,6617ae909be8b25d4c0738a7
46,50,None,6649c99486b7e549636e444a,6617ae909be8b25d4c0738a8
45,46,51,6649c99486b7e549636e444c,6617ae909be8b25d4c0738a9
54,28,55,6649c99486b7e549636e444e,664b7f7b60868f36244a77b5
57,58,None,6649c99486b7e549636e4450,6617c0fd9407da108c1fb0cb
58,59,60,6649c99486b7e549636e4452,6617c0fd9407da108c1fb0cc
61,63,None,6649c99486b7e549636e4454,6617c40c02dc207e8e3f1685
```


Figure S3: Plain text files created by VizChemoton 2.1 containing information about the compounds (A) and reactions (B) in a chemical reaction network.

InChIKey, CalcTPSA, MolLogP and MolWt), MongoDB information (basis_set, charge, energy, method, mongodb_id, multiplicity, program, solvation, and xyz coordinates) and matches in public structural databases (chebi, chembl and pubchem). Note that the RDKit properties can be dynamically demanded in the input file, and they will appear in the JSON file. The only constraint is that the properties must be written with the same name as they are called natively in RDKit. The second file corresponds to *reactions.csv*, which consists of a simple five element list for all the edges in the graph (Figure S3B). The first element is the reactant node, the second is the product node, the third corresponds to the transition state structure (barrierless reactions are depicted as `None`), the fourth is the elementary step

MongoDB ID, and the fifth is the reaction MongoDB ID.

S2 Computational Details

Chemical reaction network explorations were carried out with two different quantum chemical methods: Density Functional Theory (DFT) and Extended Tight-Binding (xTB), respectively. DFT calculations were performed with the ORCA software package version 5.0.3[3] using the long-range Perdew-Becke-Ernzerhof LC-PBE,[4–6] with the Ahlrichs def2-TZVP basis set.[7] xTB calculations were performed with GFN2-xTB[8] as implemented in SCINE xTB Wrapper version 3.0.2.[9] DFT-based CRNs were constructed in our previous work,[10] using an atom filter that limited the size of the compounds in the network to that of the ozonide; that is, the total number of atoms in the olefin plus the three oxygen atoms from ozone. In this work, CRNs constructed with xTB were calculated without limiting the size of the compounds during the exploration (i.e., no atom filter was applied), in order to avoid disregarding important diol compounds occurring during ozonation, as discussed in Petrus et al.[10]

Table 1 in the main manuscript shows the computational costs associated to the construction of the CRNs as serial computing time, which corresponds to the time invested in all the calculations of the CRN on a hypothetical single processor core.[11] Because SCINE enables CRN explorations to run in parallel, the effective computing time (i.e., wall clock time) results from dividing the serial computing time by the number of cores employed. Since we employed 250 cores for DFT CRNs and 1000 cores for xTB CRNs, the wall clock time was considerably reduced. xTB provides a 10-100 speed up factor with respect to the use of DFT, consistent with previous benchmarks.[12] Table 1 also describes the size of the CRNs in terms of number of reactions, compounds, and flasks (i.e., non-bonded complexes in SCINE notation). The total number of compounds in the SCINE CRNs is determined by Molassembler[13] version 2.0.1[14] which assigns unique IDs to every new compound in the database. Unless otherwise stated, we implicitly account for flasks when referring to com-

pounds. Because of approximate 10-fold smaller computing times, xTB-CRNs are substantially larger than those constructed with DFT. xTB-CRNs include 40000-80000 compounds and 80000-120000 reactions, whereas DFT-CRNs contain 400-1800 compounds and 500-6000 reactions. Note that the number of compounds and reactions for DFT CRNs discussed here differs slightly from the ones reported originally by Petrus et al.[10] because we only account for the results obtained in the exploration phase of CRN construction.

S3 Conversion of Compounds to SMILES/RDKit Mol Objects

S3.1 RDKit mol object conversion

For the conversion of QC structures to RDKit mol objects, we used the LC-PBE and GFN2-xTB bond orders calculated automatically during the SCINE reaction explorations (henceforth, SCINE-BOs). These QC bond orders are computed on-the-fly as Molassembler exploits this information to distinguish between novel and repeated compounds. To evaluate the performance of SCINE-BOs, we have considered a second RDKit mol object conversion method. It relies on a rule-based prediction of bond orders developed by Kim and Kim[15] and implemented in RDKit as xyz2mol.[16] Table 1 in the main manuscript summarizes the RDKit mol object conversion performance using SCINE-BOs and xyz2mol for the four distinct CRNs considered in this study. The percentage of compounds that were successfully converted with SCINE-BOs and xyz2mol ranged from 62% to 94% and 60% and 74%, respectively. While xyz2mol performs better for CRNs calculated with DFT than SCINE-BOs, the latter excels for CRNs calculated with xTB. The data in Table 1 also show that a considerable share of compounds (up to 40%) for both CRNs-E and CRNs-T were not successfully converted to RDKit mol objects.

We inspected failed conversion cases of xyz2mol and SCINE-BOs based on the com-

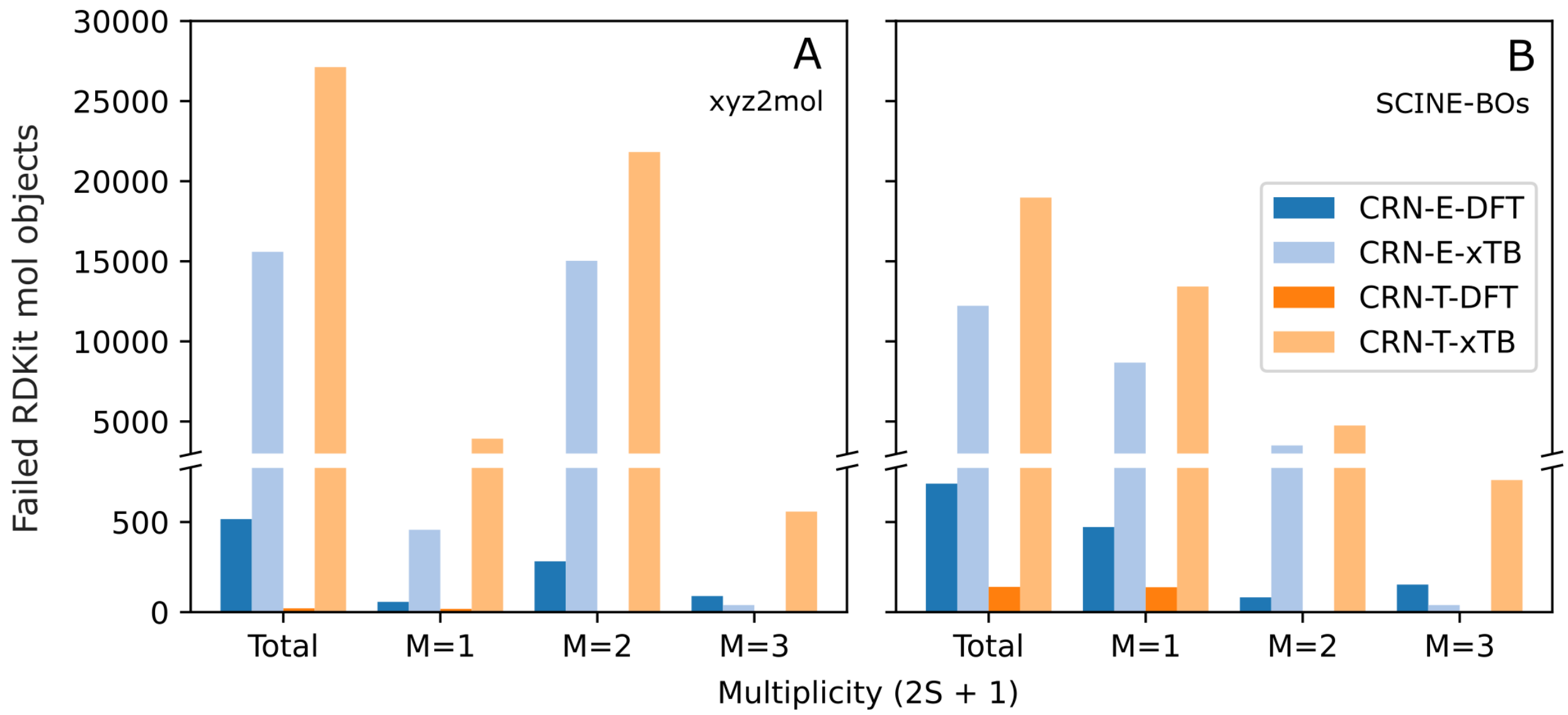

Figure S4: Number of compounds in the four CRNs that could not be converted to RDKit mol objects using xyz2mol (A) and SCINE-BOs (B). Compounds are sorted according to the spin multiplicity: singlets (M=1), doublets (M=2) and triplets (M=3).

pound's multiplicity, given that string molecular representations lack consideration of the spin states. Figures S4 and S7 show the total number of compounds that could not be converted to RDKit mol objects, and the respective shares according to their multiplicity. If the total number of failed cases per CRN are considered, xyz2mol slightly outperforms SCINE-BOs for the small DFT CRNs. The reverse observation is made for the large xTB-CRNs, with fewer failed cases per CRN for SCINE-BO. For example, conversion with xyz2mol results in 15592 and 27122 failed cases for CRN-E-xTB and CRN-T-xTB, respectively, while failed cases with SCINE-BOs amount to 12222 and 18978, respectively. The reason for these trends becomes apparent when comparing the number of failed cases with their multiplicity (singlets vs. spin-unpaired doublets and triplets). xyz2mol generally performs equally well or better than SCINE-BO, except for spin-unpaired doublets (Figure S9). Indeed, most of the SCINE-BOs failed singlet cases in Figure S4B were correctly assessed by xyz2mol (see Figure S5). This subset of compounds consisted of zwitterions in which positive and negative formal charges were frequently located on oxygen and carbon atoms (see Figure S6). These zwitterionic structures are correctly converted by xyz2mol, which assigns formal

charges to atoms in charged fragments that do not meet standard neutral valences. SCINE-BOs, in contrast, do not consider predefined chemical rules and therefore do not represent these charge-separated structures adequately. The performance of SCINE-BOs is superior once structures with unpaired electrons are considered (Figure S9, M=2). For this subset of doublets, failed conversion with xyz2mol reach up to 80%. This observation is a direct consequence of the lack of spin information in the xyz2mol framework, which only considers charge and cartesian coordinates.

S3.2 Singlet compounds: SCINE-BOs vs xyz2mol

When SCINE-BOs were used to convert singlet compounds to RDKit mol objects, an anomalous high failed-to-succeeded conversion was observed (see Figure S4 in the main text). This result was in stark contrast with the high conversion ratio of xyz2mol for singlet compounds. To investigate the origin of the issue, we selected the subset of compounds that could not be converted to RDKit mol objects using SCINE-BOs, and compared them one-to-one with the results generated with xyz2mol. Figure S5 shows the singlet compound RDKit mol object conversion using SCINE-BOs (red color) and xyz2mol (green color). It became clear that xyz2mol outperforms SCINE-BOs, converting over 97% of the failed cases. To determine the origin of this large difference, we analyzed the chemical nature of this subset of singlet compounds.

Over 95% of this subset consist of zwitterionic compounds, which include positive formal charges on oxygens and negative formal charges on carbons. Figure S6 shows a sample of six singlet compounds which were not converted to RDKit mol objects with SCINE-BOs, but they were with xyz2mol. Notice that ball-and-stick molecular representations are plotted from the original cartesian coordinates collected in the CRNs, hence no bond-type is assumed. Figure S6A depicts a compound where C1 has a negative formal charge and O1 (part of a hydroxide functional group) has a positive formal charge. Figure S6B depicts a similar case as the latter, since C2 has a negative formal charge and O5 has a positive formal charge.

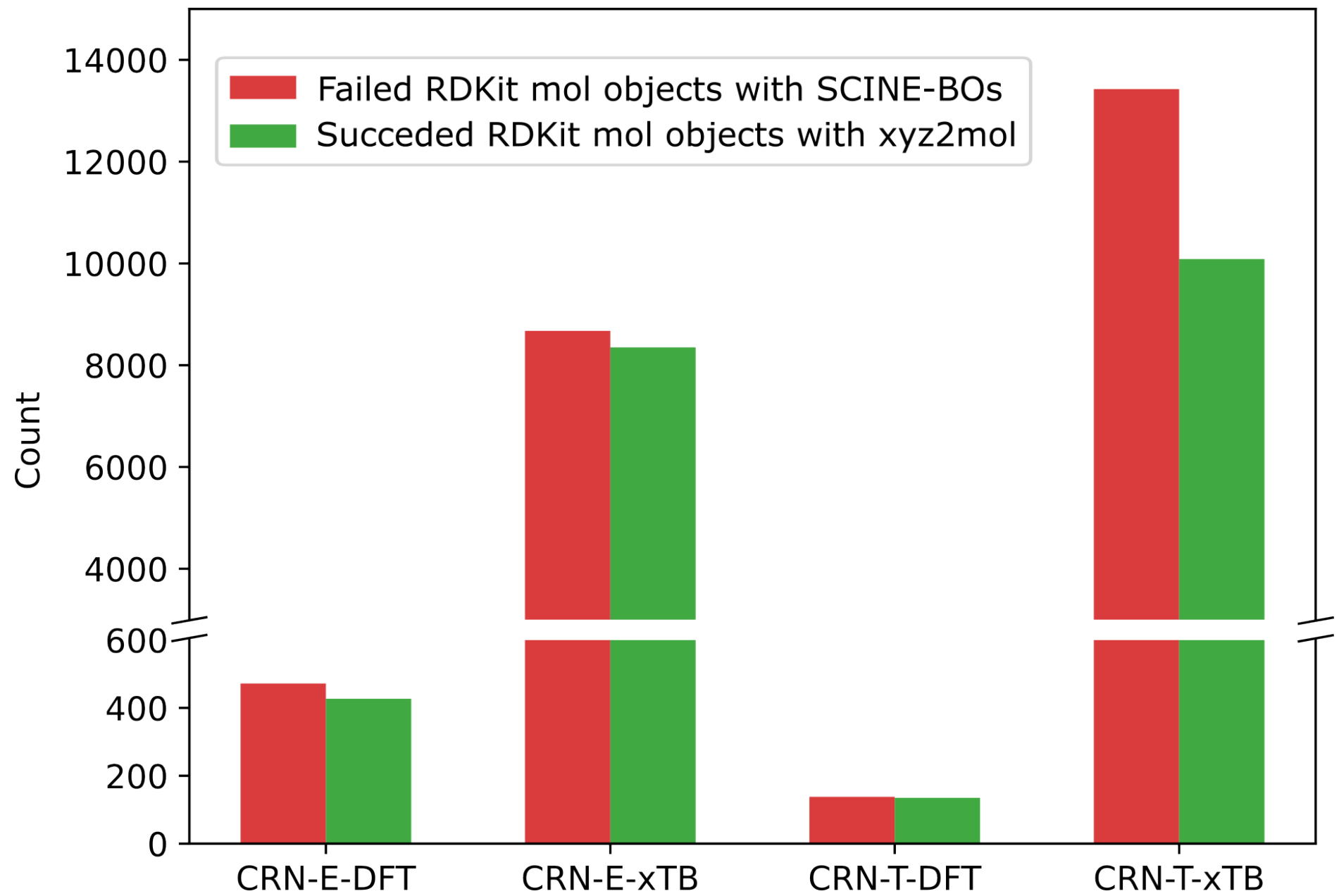

Figure S5: Conversion of singlet compounds to RDKit mol objects using SCINE-BOs (red color) and xyz2mol (green color) for four CRNs constructed with Chemoton.

Figure S6C shows a new case, since O1 (part of an aquo functional group) has a positive formal charge and C1 has a negative formal charge. Figure S6D shows two double-bond compound, where O5 has a positive formal charge, and O4 has a negative formal charge. Figure S6E shows a compound with a double bond between C2 and O4, where C2 has a formal negative charge and O4 a formal positive charge. Figure S6F shows a compound where the formal charges are in oxygen atoms O3 (positive) and O4 (negative), respectively. The six cases described here highlight that xyz2mol successfully tackles the conversion of zwitterion compounds to RDKit mol objects, regardless of which atom types (oxygen or carbon) present the formal charges.

We also explored the subset of singlet compounds that could not be successfully converted to RDKit mol objects with xyz2mol. Of the 458 failed cases, 357 (78%) correspond to flasks. Figure S7 shows six examples that demonstrate the type of flasks that were not captured with xyz2mol. Figure S7 on top shows three cases in which molecular hydrogen is formed. Figures S7 below show three cases in which water or hydroxyls are part of the flask.

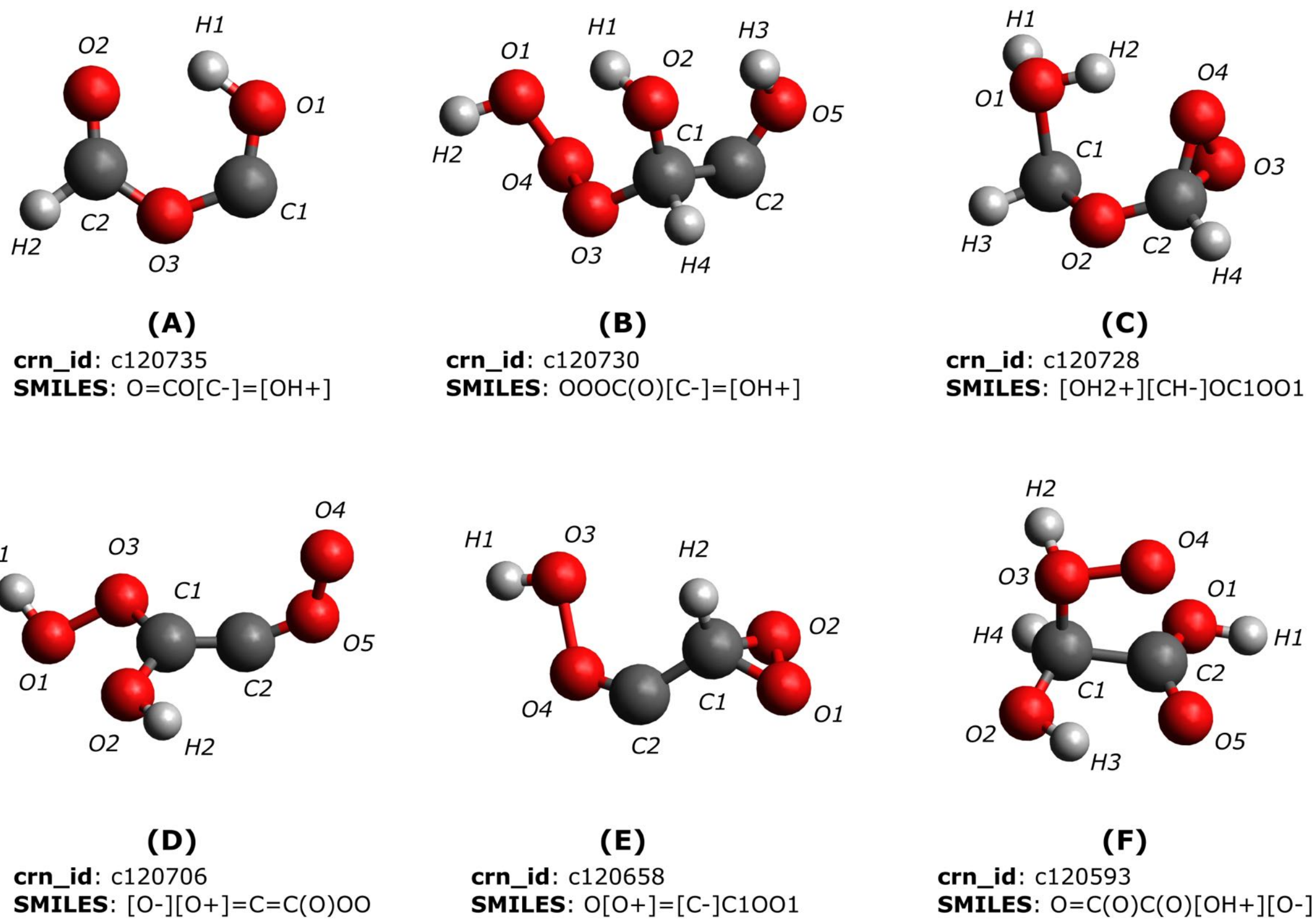

Figure S6: Sample of six compounds in CRN-E-xTB which could not be converted to RDKit mol objects using SCINE-BOs. Molecular structures represent the original cartesian coordinates, thus all bonds are represented as single-bonds. SMILES were obtained from the RDKit mol objects generated with xyz2mol.

S3.3 Non-singlet compounds: SCINE-BOs limitations

Given that xyz2mol does not account for multiplicity when converting a molecular structure to SMILES, non-singlet compounds should be converted using SCINE-BOs. Although Figure S4 in the main text shows that SCINE-BOs perform well when converting non-singlet compounds to RDKit mol objects, we analyzed which compounds could not be converted successfully. In CRN-E-xTB there are a total of 3548 non-singlet compounds that were not converted to RDKit mol objects with SCINE-BOs, of which 2135 correspond to flasks, that is, 60%. We also observed this trend in section S3.2, where more than half of the failed cases correspond to flasks. Figure S8 shows six examples that demonstrate the chemical complexity that was not captured with SCINE-BOs. Figure S8 shows three doublet cases

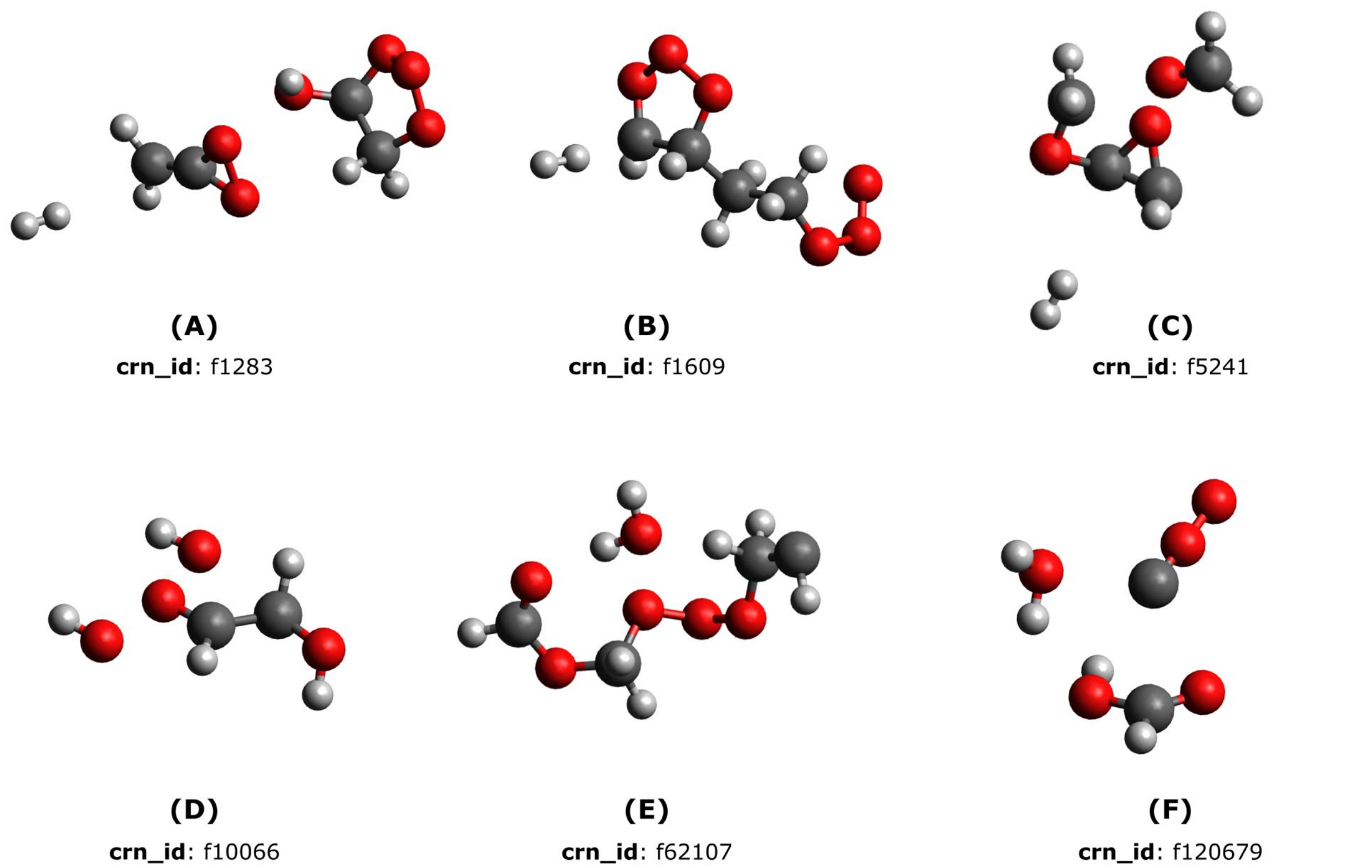

Figure S7: Sample of six flasks in CRN-E-xTB which could not be converted to RDKit mol objects using xyz2mol. Molecular structures represent original cartesian coordinates.

and three triplet cases for the top and bottom parts, respectively.

S3.4 Hybrid approach: SCINE-BOs and xyz2mol

We observed that SCINE-BOs performs well for non-singlet multiplicities, but it does not with zwitterion compounds (Figure S4 of the main text). In contrast, we observed that xyz2mol performs well for singlet compounds –zwitterion in specific– but it underperforms for non-singlet multiplicities. This pattern can be observed in more detail in Figure S9A and Figure S9B, respectively. Notice that both plots represent the same data, but at different ranges to facilitate their interpretation. Singlets (circle markers) are systematically on the top half of the plot, whereas doublets (diamond markers) are systematically on the bottom half of the plot. Therefore, we evaluated a third approach, which we refer to as hybrid, in which, first, compounds are converted to RDKit mol objects with SCINE-BOs. If conver-

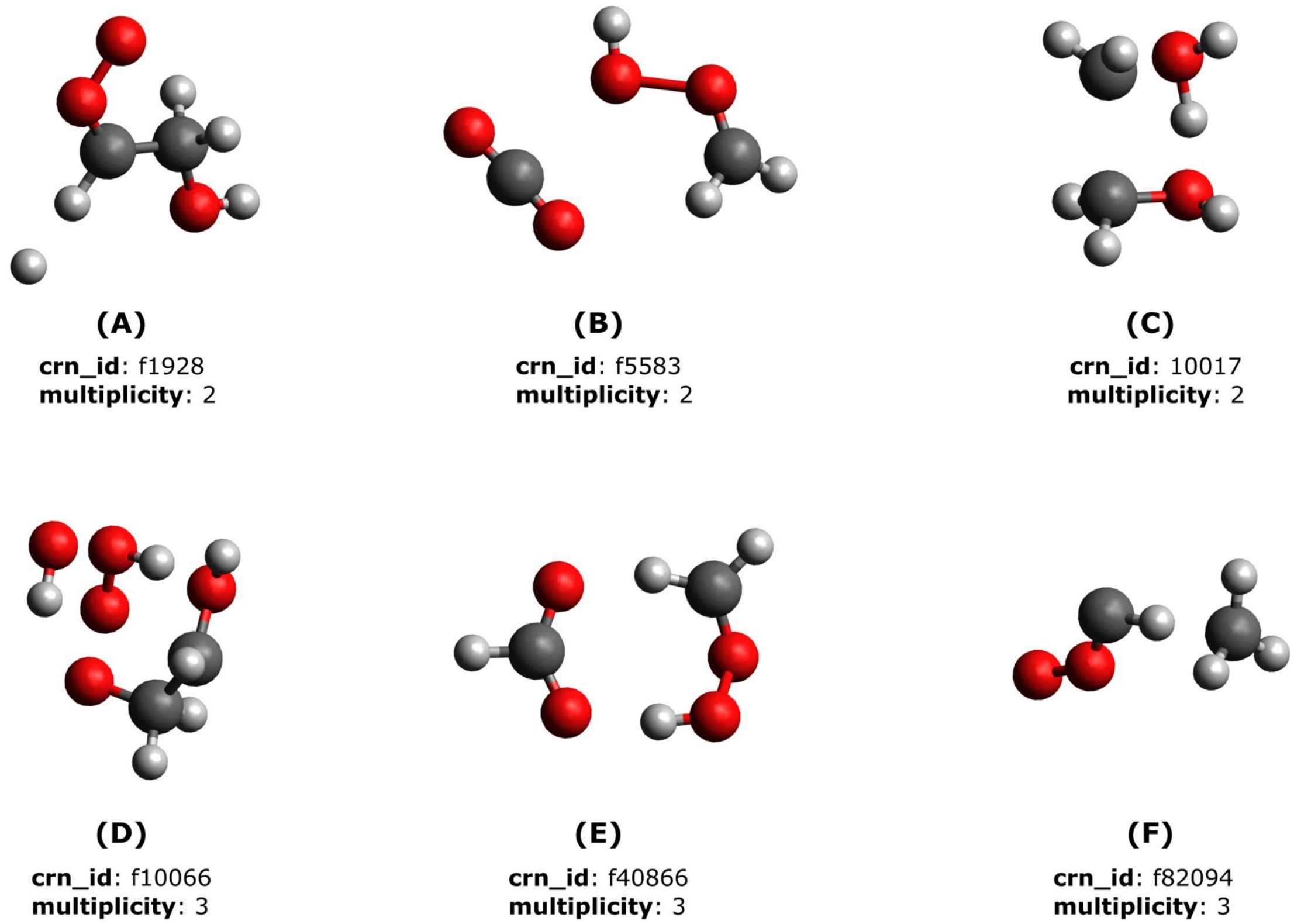

Figure S8: Sample of six flasks in CRN-E-xTB which could not be converted to RDKit mol objects using SCINE-BOs. Molecular structures represent original cartesian coordinates.

sion fails and the compound is a singlet, xyz2mol is used instead. Figure S9C shows the performance of the three methods in terms of the total number of failed conversions of compound to RDKit mol object. We observed that the hybrid approach outperforms xyz2mol and SCINE-BOs for both DFT and xTB CRNs. Therefore, we defined the hybrid approach as the default method to obtain SMILES and InChIKeys in the SCINE CRNs.

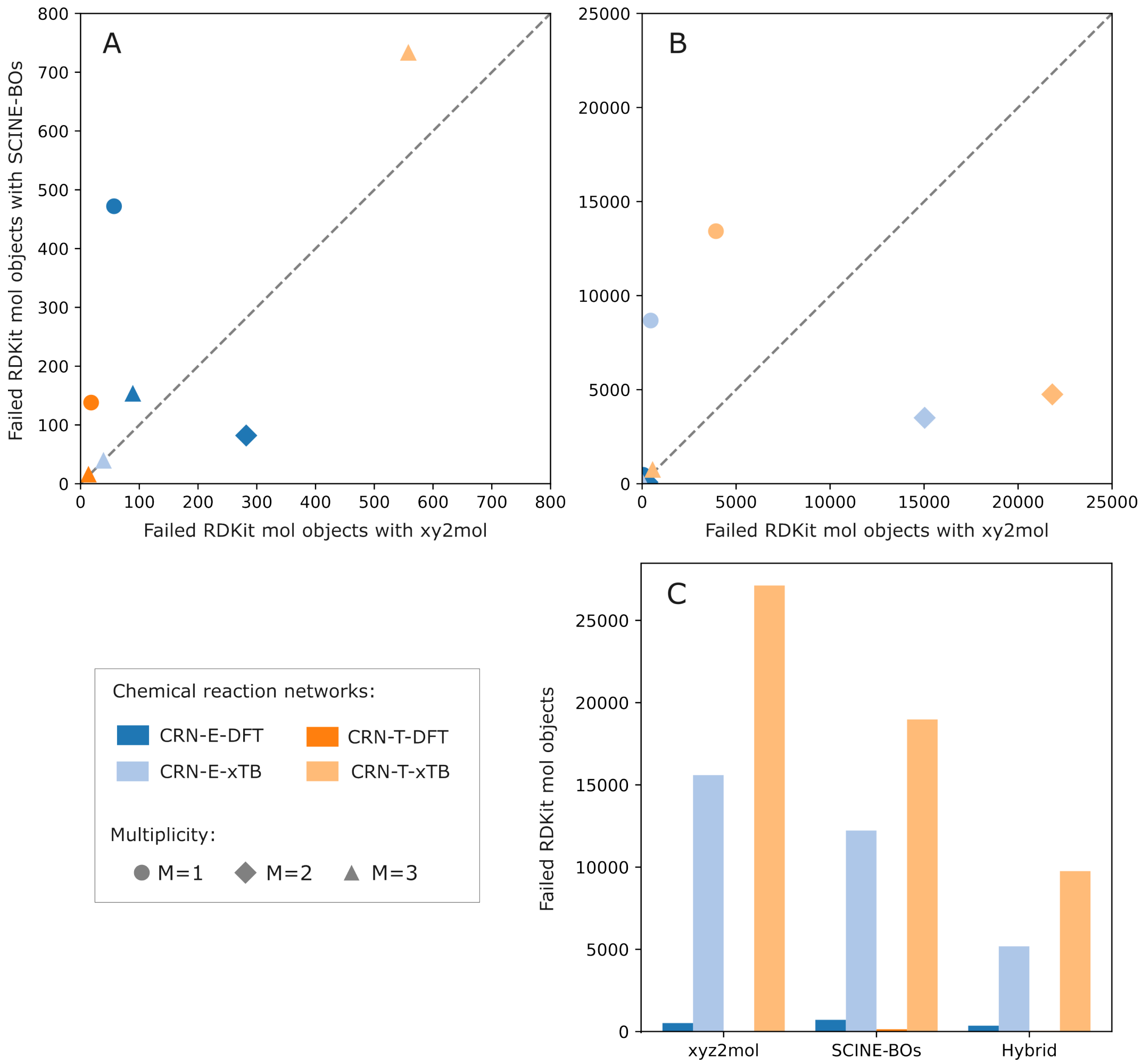

Figure S9: Number of compounds that could not be converted to RDKit mol objects. Scatter plots between the failed singlet, doublet and triplet cases using xyz2mol and SCINE-BOs, respectively, for the segment (A) and the total number (B). Bar plot indicating the total number of failed cases for xyz2mol, SCINE-BOs and Hybrid.

S4 Application Examples

S4.1 DFT and xTB compound chemical spaces

Once the compounds of the four CRNs were converted to RDKit mol objects, it became possible to determine the common InChIKeys between the DFT and xTB networks. We refer to this study as a comparison between *compound chemical spaces*, although we should emphasize that this space only represents the subset of compounds that could be converted to RDKit mol objects. Table 1 in the main text shows that CRN-E-DFT shared 443 compounds with CRN-E-xTB, and CRN-T-DFT shared 220 compounds with CRN-T-xTB.

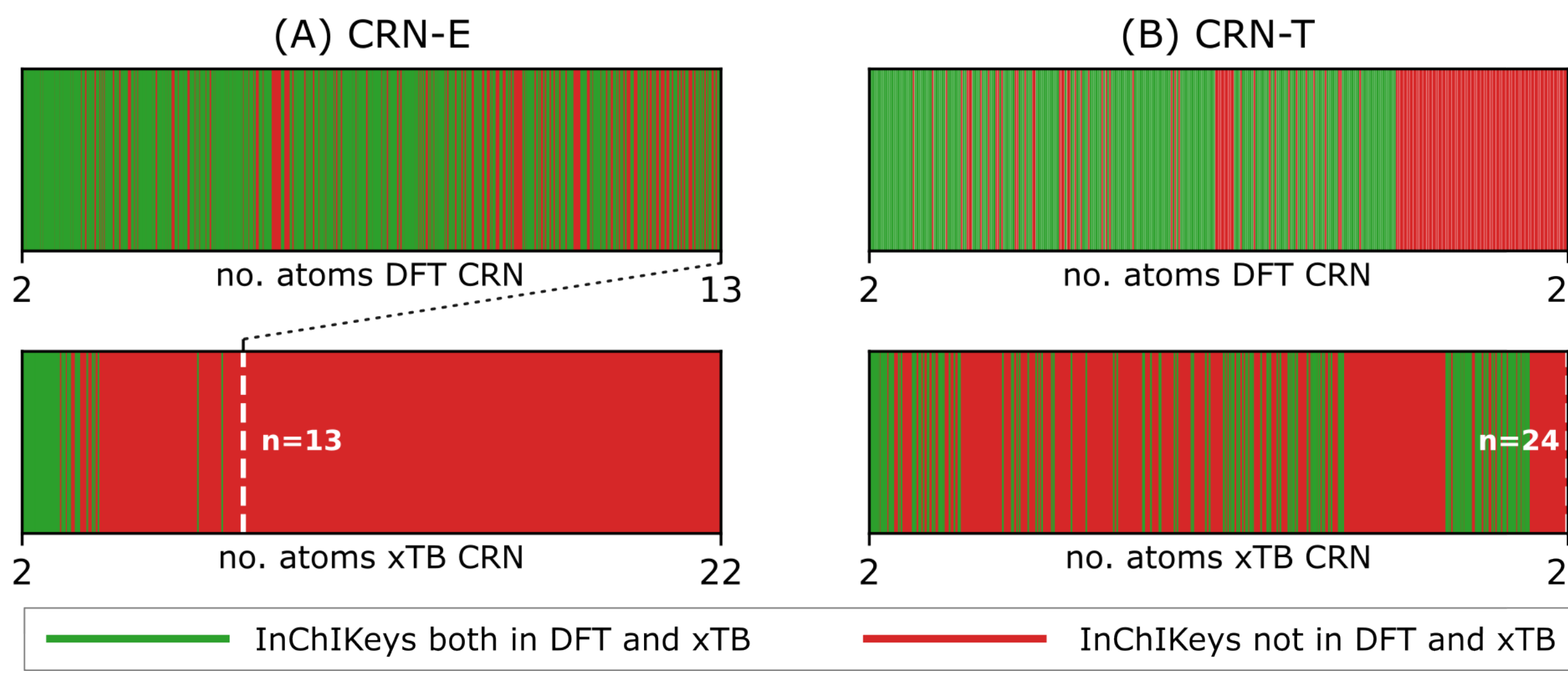

Figure S10: Barcodes for the common compound chemical space between xTB and DFT networks for (A) CRN-E and (B) CRN-T. Green lines indicate overlap between xTB and DFT InChIKeys entries; red lines indicate presence in only one dataset. Compounds were converted to RDKit mol objects with the Hybrid method, and subsequently to InChIKeys.

Figure S10 shows two pairs of barcodes that depict the compound chemical space shared between DFT and xTB networks. The x-axis represents the total number of unique InChIKeys for a given CRN, sorted according to the total number of atoms. Green colored lines indicate that an InChIKey is common for a pair of DFT and xTB networks, whereas red colored lines indicate that an InChIKey is only found in one of the two networks. Figure S10A shows the 443 common InChIKeys between DFT and xTB CRNs-E in two different plots. On

the one hand, Figure S10A top shows that most of the InChIKeys in DFT CRN-E are also present in the xTB network. On the other hand, Figure S10A bottom demonstrates that the InChIKeys from the DFT network account for a small proportion of the xTB compound space. Figure S10B shows the 220 common InChIKeys between DFT and xTB CRNs-T in two different plots. Most of the xTB InChIKeys are present in Figure S10B top, whereas the DFT InChIKeys cover only a small proportion of the xTB compound space. In both cases, xTB networks cover most of the compound chemical space found by DFT, while maintaining a lower computational cost, thus strengthening our decision to perform the exploratory phase with this tight-binding method.

S4.2 Public structural databases

Figure S11 shows the number of InChIKeys that were found in PubChem for the four CRNs considered in this study. The data is also reported in Table 1 of the main manuscript.

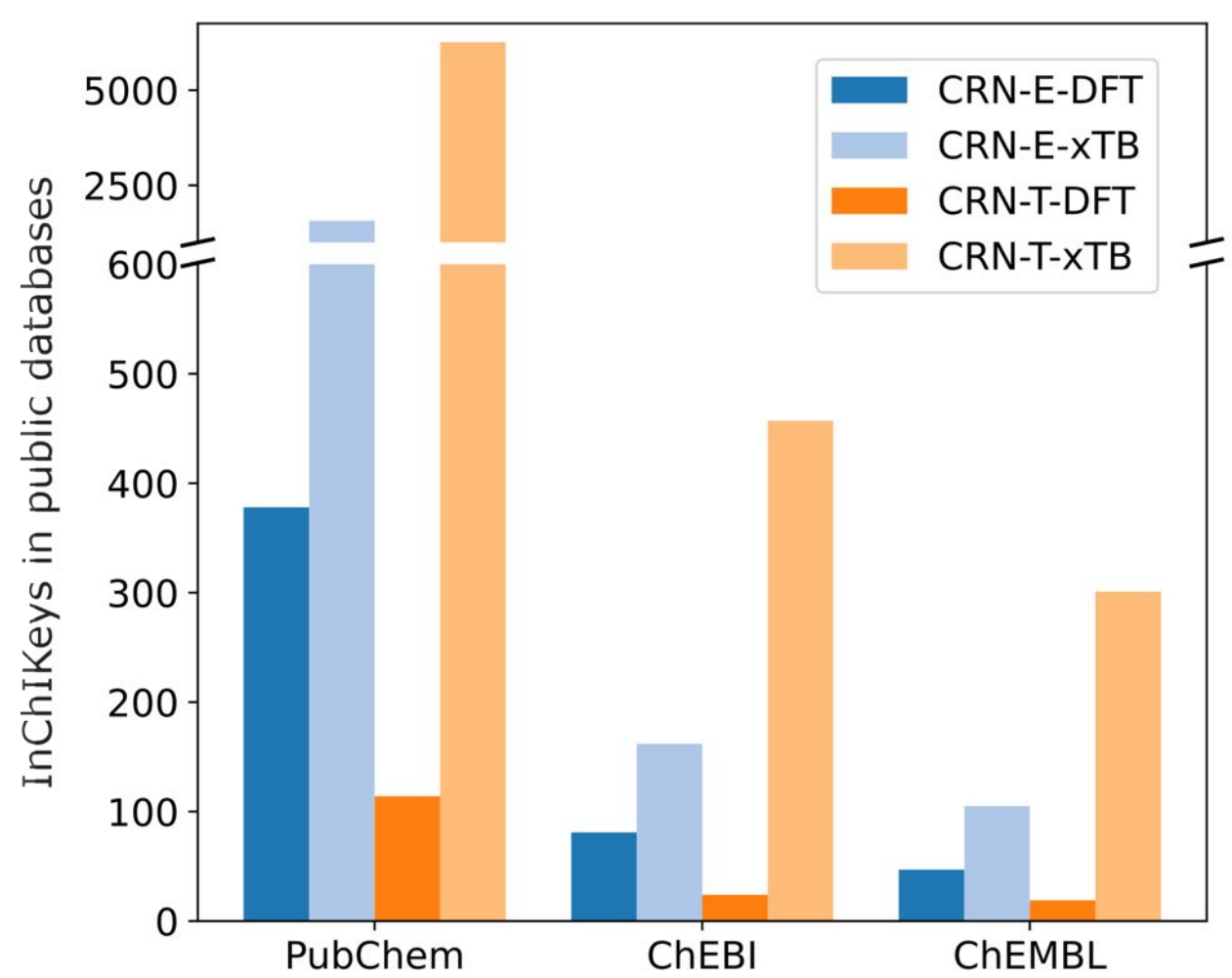

Figure S11: Number of InChIKeys in the four CRNs considered in this study that are also reported in PubChem, ChEBI and ChEMBL.

S4.3 Chemical property descriptors

Figure S12 shows three cheminformatic property distributions, available in RDKit, for xTB CRNs.

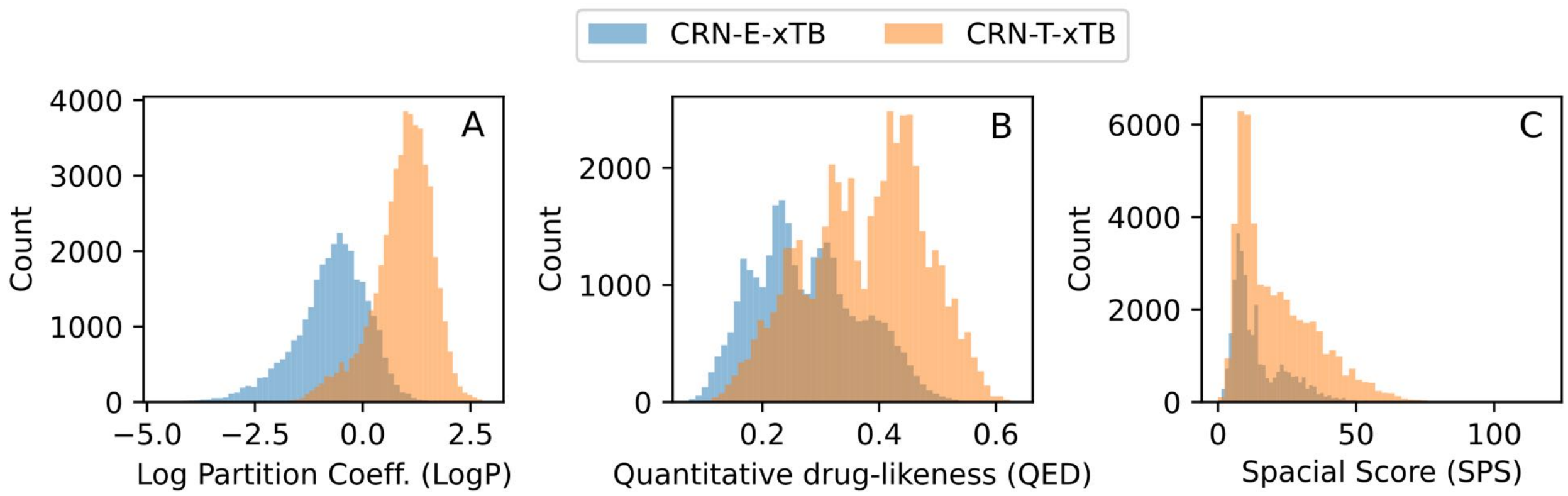

Figure S12: Histograms for three cheminformatic property distributions: partition coefficient (A), quantitative drug-likeness (B), and spacial score (C) for CRN-E-xTB (blue color) and CRN-T-xTB (orange color), respectively.

S4.4 Machine learning

Figure S13 shows a downstream machine learning application with the interoperable data generated with VizChemoton to annotate MS^2 spectra. As an example, we imported the unannotated MS^2 spectra of 2-pentanone[17,18] into Sirius[19] to determine the molecular formula and calculate the CSI:FingerID fingerprint, which is later used in the structure elucidation step. Next, we converted the compound JSON file of CRN-T-xTB to a TSV, containing all the SMILES in the CRN, and imported it as a custom database using this available feature in Sirius. When DSSTox and PubChem databases were used, the predicted structure showed a lower Tanimoto similarity (0.0930) than when the CRN-T-xTB structural database was employed (0.2000). Although the current application represents only a minimal example, it showcases the potential of leveraging quantum reaction data in other fields such as cheminformatics and machine learning.

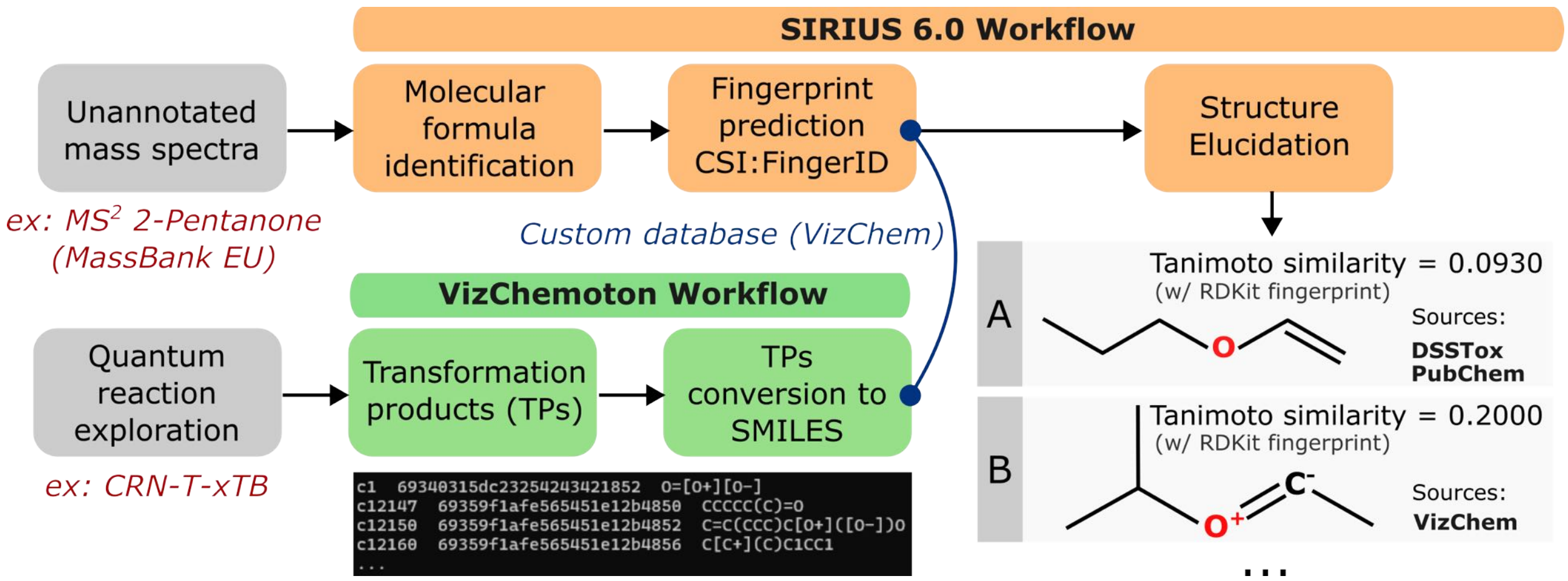

Figure S13: Workflow for leveraging the quantum reaction data generated with Chemoton to enhance the annotation of MS^2 spectra using the machine learning based method Sirius. Tanimoto similarity was calculated using the RDKit fingerprints and using the 2-pentanone as reference structure.

S5 Visualization Features

S5.1 General layout

Figure S14 shows the default view of the HTML layout generated with VizChemoton, which depicts the chemical reaction network on the left, ball-and-stick representation of the selected node in the network, on the right, and a 2D integrated interface to generate SMILES and InChIKeys to ease the interaction with the reaction network, at the bottom.

The layout can be divided in three parts. The left part concerns the display and interaction with the chemical reaction network, which has multiple buttons for (1) moving the network, (2) zooming in a given part of the network, (3) resetting the view of the network, (4) nodes and edges of the network that can be pressed, (6) a placeholder for text input, (7) a button to locate a certain node according to the text introduced on the left, (8) a button to highlight the reaction neighbors of the selected molecule, (9) a button to hide the barrierless reactions, and (10) a button to isolate a node from the rest of the network. The right part displays the selected nodes or edges to (5) visualize the compounds, flasks and transition states. The ball-and-stick visualization is run by JSMol.js, therefore, it is neces-

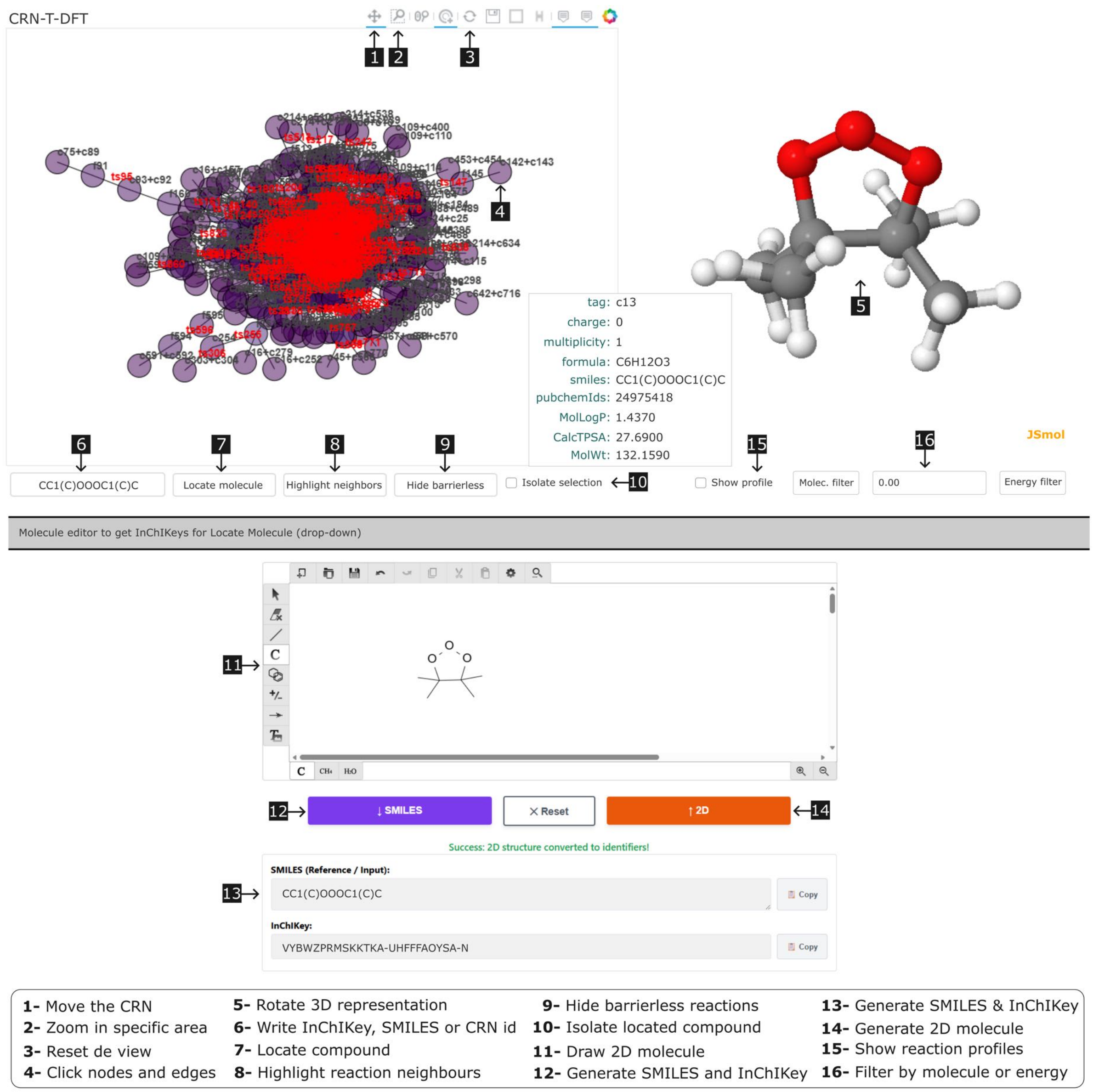

Figure S14: Complete overview of the VizChemoton HTML dashboard of CRN-T-DFT.

sary to have internet access so that this javascript module is automatically loaded (this is also the case for the next two javascript libraries employed). If the user specified to calculate the reaction profiles (between a source and a target compound) in the YAML file, it will be possible to visualize the reaction profiles (15) and filter those profiles based on specific molecules and/or energy thresholds (16). An example of the use of reaction profiles is given in Figure S18 The bottom part has a 2D molecule editor powered by Kekule.js which allows sketching a molecule of interest (11). It is possible to convert the 2D molecule to SMILES

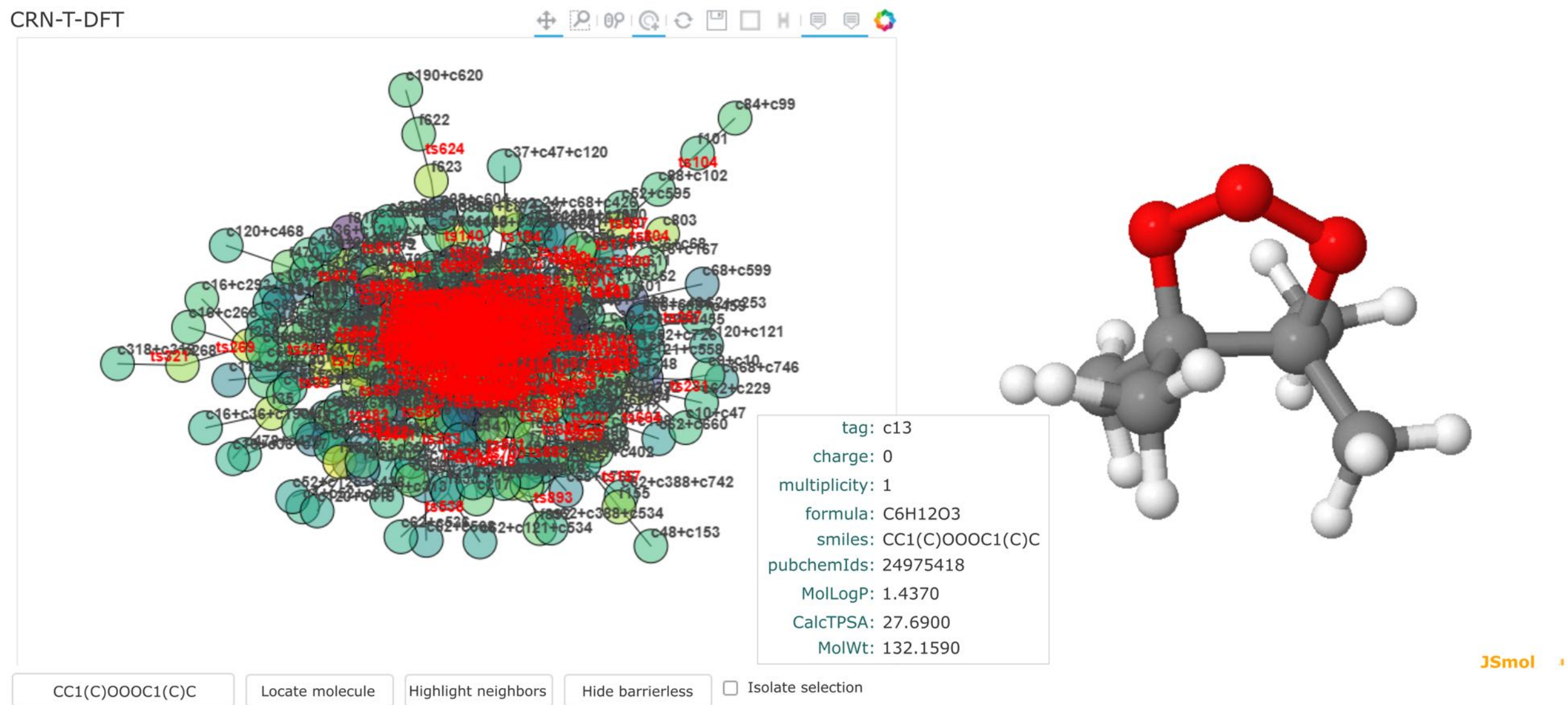

Figure S15: Quantitative view of the logP using the viridis color scale on the HTML dashboard of CRN-T-DFT.

and InChIKey format (12) using RDKit.js and copy the string-representation (13) so that the user can input it in the text input above (7). Alternatively, once can copy a SMILES in (13) and generate the 2D structure using (14). On a technical note, the top and bottom parts of the HTML are independent (i.e. belong to two sandboxes) to avoid incompatibilities between Bokeh.js/JSMol.js and Kekule.js/RDKit.js.

S5.2 Quantitative mapping

It is possible to map chemical properties of the CRN in the HTML layout, in order to ease the interpretation of the CRN. Figure S15 depicts a CRN where the partition coefficient (logP) values are coloured on top of the nodes based on the viridis color scale. Dark purple color corresponds to low logP, whereas light green color corresponds to large logP.

S5.3 Large chemical reaction networks

DFT reaction networks are small-to medium-sized (a few hundred nodes); therefore, it is not computationally expensive to find an optimal arrangement for the visualization of the

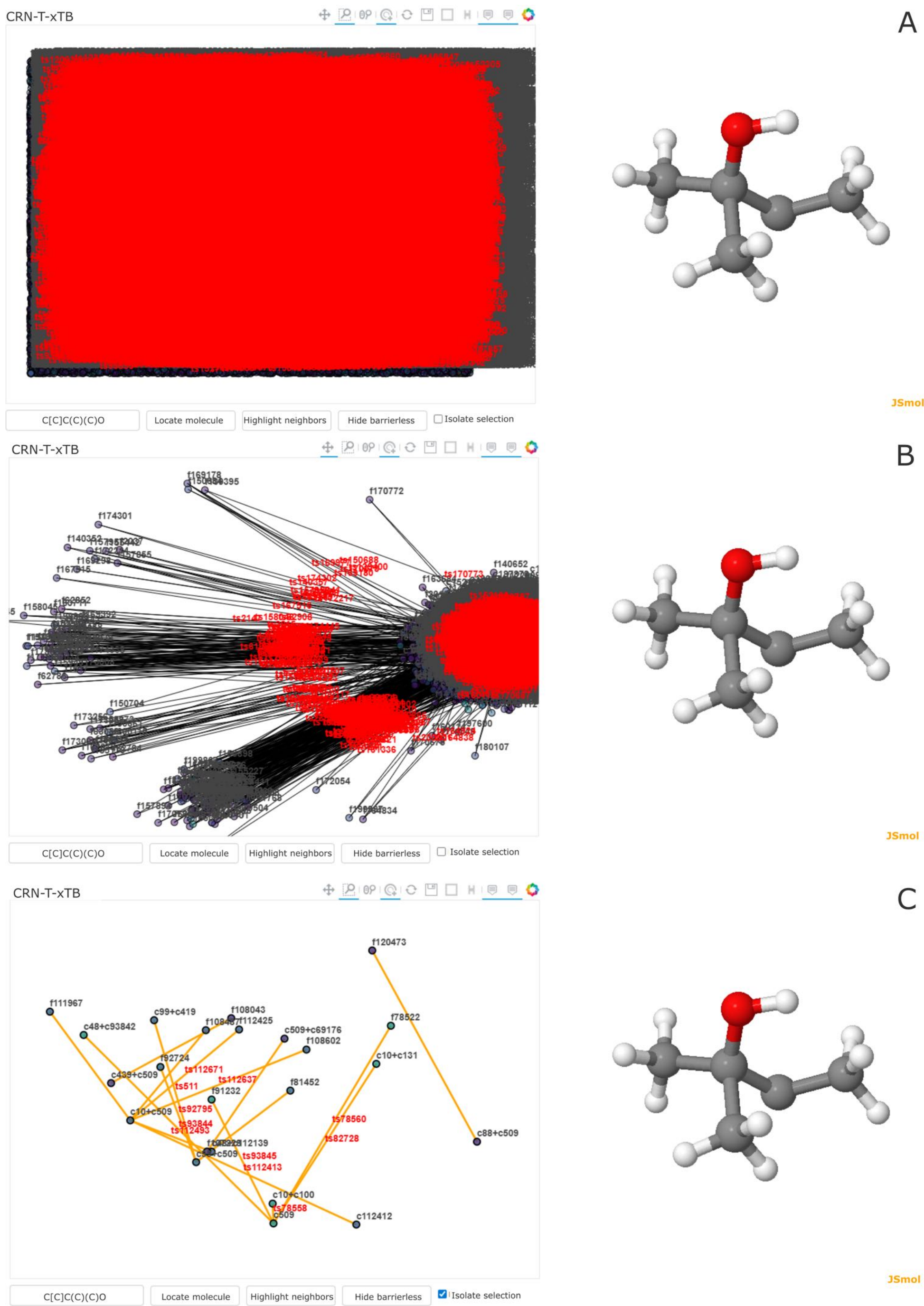

Figure S16: HTML dashboard for CRN-T-xTB centered at `C[C]C(C)(C)O` with (A) a random distribution of nodes and edges, (B) clustered distribution of nodes and edges using k-means, and (C) reduced view using the isolate selection feature based on the first reaction layer.

network. We use as a default method the Kamada Kawai[20] and Fruchterman-Reingold[21] algorithms, as implemented in the Python library, NetworkX.[22] However, xTB reaction net-

works are one-to-two orders of magnitude larger than DFT networks (see Table 1 of the main article), so Kamada Kawai (and similar methods such as FruchtermanReingold) become impractical due to large memory usage. The default option for xTB CRNs is to randomly distribute the nodes and edges of the network in the HTML layout (Figure S16A). However, this makes it very difficult to interact with the network as: first, the nodes and edges are overlapped, and second, nodes connected by a same edge can be set very far apart in the canvas. To address this issue in a scalable manner, we have implemented a k-means clustering[23] to set similar molecular structures closer on the canvas (Figure S16B). To determine the optimal number of clusters on-the-fly, we implemented the silhouette method.[24] As a measure of chemical similarity, we relied on four basic descriptors derived from the cartesian coordinates of the molecular structures: total number of atoms, total number of heavy atoms, radius of gyration, and volume. We chose these basic parameters, instead of SMILES-derived parameters, because not all compounds in the CRNs could be converted to SMILES. Therefore, the choice of plain descriptors represents a simple yet robust option. Once the distribution of nodes and edges is more chemically-intuitive than the random distribution, we tackled the pruning of the network, so that the user can focus on a specific compound and its reactions. The `isolate selection` feature allows one to select the compound of interest via SMILES, MongoDB id or CRN id, followed by selecting the nearest neighbors, and ending with pressing the isolate selection (Figure S16C). This short protocol, combined with the k-means clustering enables a meaningful interaction with very large CRNs.

S5.4 Qualitative mapping

Figure S17 depicts CRN-T-DFT centered on the ozonide and with the two adjacent reaction layers, showcasing the use of a qualitative mapping to indicate PubChem coverage with the reaction network.

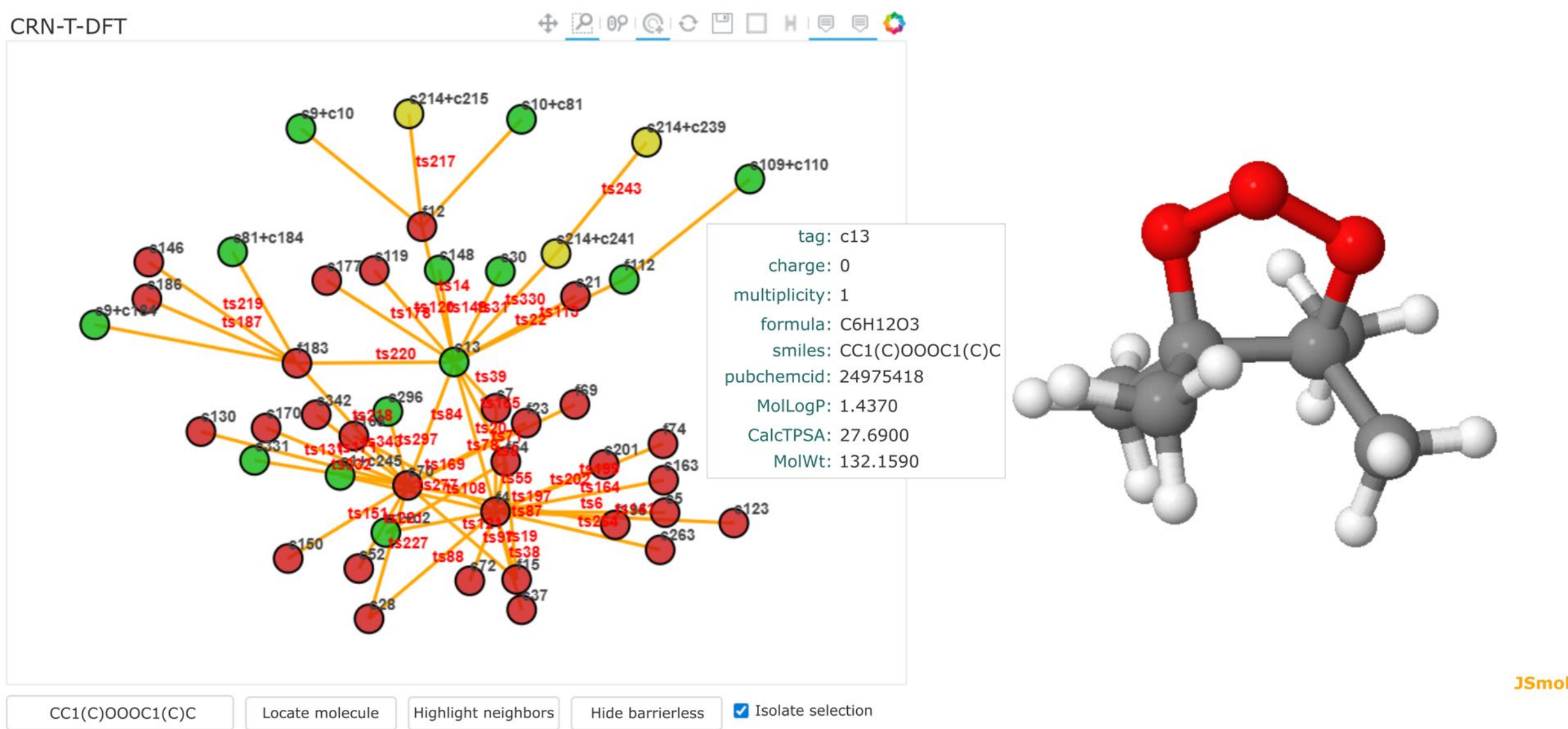

Figure S17: Qualitative view of the PubChem coverage of compounds within each node: both compounds are present (green), only one compound is present (yellow), and neither compound present (red) for CRN-T-DFT centered on the ozonide and with the two adjacent reaction layers.

S5.5 Reaction profiles

Figure S18 depicts CRN-T-DFT, showcasing the five shortest reaction pathways between c1 (ozone) and c48 (formaldehyde). Reaction profiles are determined using a depth-first search algorithm[25] together with the Pathfinder compound costs. When a large number of reaction profiles are generated, they can be filtered using either the molecular filter, which selects profiles containing a specified compound or flask, or the energy filter, which excludes profiles above a user-defined energy threshold.

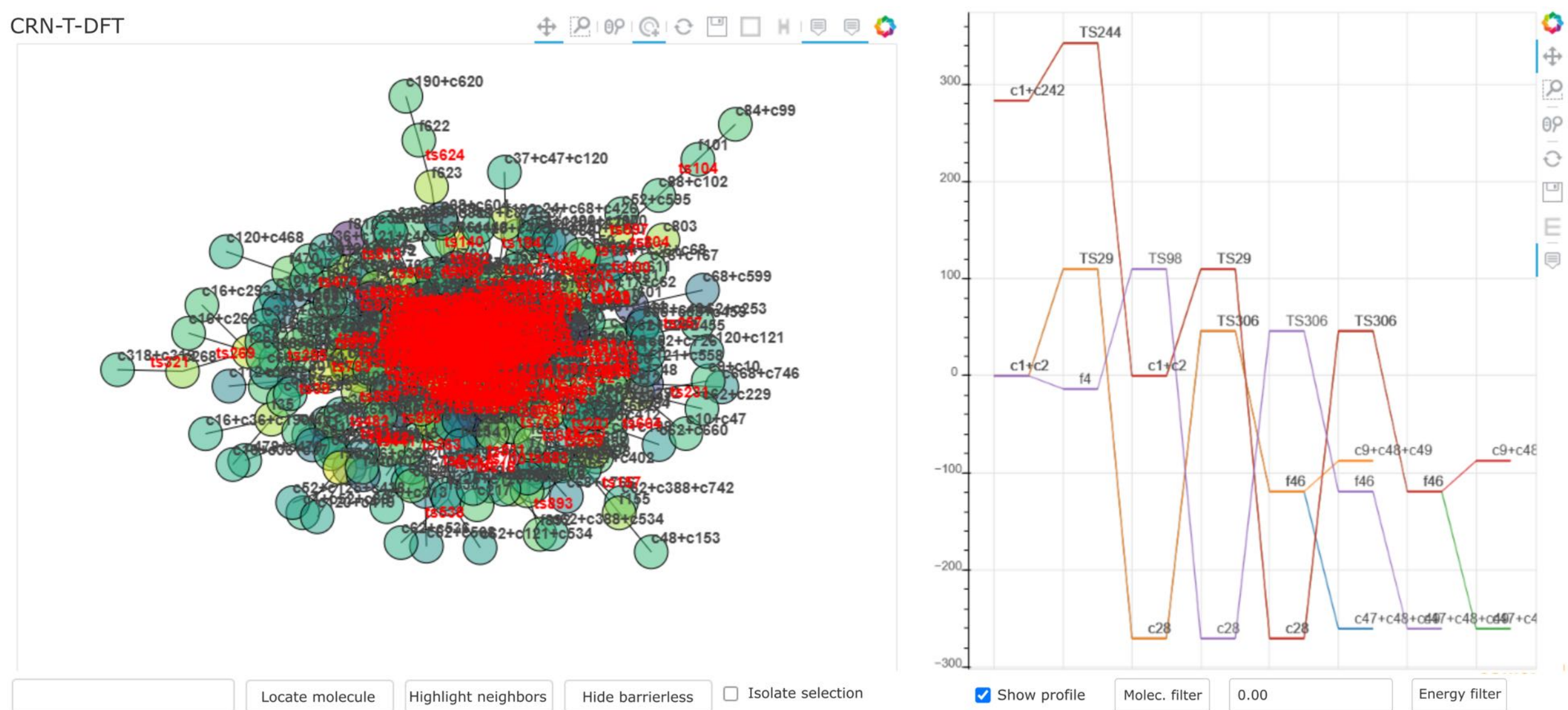

Figure S18: HTML dashboard showing the five shortest reaction pathways between ozone and formaldehyde.

References

(1) Weymuth, T.; Unsleber, J. P.; Türtscher, P. L.; Steiner, M.; Sobez, J.-G.; Müller, C. H.; Mörchen, M.; Klasovita, V.; Grimmel, S. A.; Eckhoff, M.; Csizi, K.-S.; Bosia, F.; Bensberg, M.; Reiher, M. SCINE—Software for chemical interaction networks. *J. Chem. Phys.* **2024**, *160*, 222501, https://doi.org/10.1063/5.0206974.

(2) ETH Zurich, Department of Chemistry and Applied Biosciences, Reiher Group VizChemoton Documentation: Input file. version 1.0.0, ETH Zurich, 2026; Accessed: 2026-07-20.

(3) Neese, F. Software update: the ORCA program system—Version 5.0. *WIREs Comput Mol Sci.* **2022**, *12*, e1606, https://doi.org/10.1002/wcms.1606.

(4) Perdew, J. P. Density-functional approximation for the correlation energy of the inhomogeneous electron gas. *Phys. Rev. B* **1986**, *33*, 8822–8824, https://doi.org/10.1103/PhysRevB.33.8822.

(5) Perdew, J. P. Erratum: Density-functional approximation for the correlation energy of the inhomogeneous electron gas. *Phys. Rev. B* **1986**, *34*, 7406–7406, https://doi.org/10.1103/PhysRevB.34.7406.

(6) Vydrov, O. A.; Scuseria, G. E. Assessment of a long-range corrected hybrid functional. *J. Chem. Phys.* **2006**, *125*, 234109, https://doi.org/10.1063/1.2409292.

(7) Schäfer, A.; Huber, C.; Ahlrichs, R. Fully optimized contracted gaussian basis sets of triple zeta valence quality for atoms Li to Kr. *J. Chem. Phys.* **1994**, *100*, 5829–5835, https://doi.org/10.1063/1.467146.

(8) Bannwarth, C.; Caldeweyher, E.; Ehlert, S.; Hansen, A.; Pracht, P.; Seibert, J.; Spicher, S.; Grimme, S. Extended tight-binding quantum chemistry methods. *WIRES Comp. Mol. Sci.* **2021**, *11*, e1493, https://doi.org/10.1002/wcms.1493.

(9) Bensberg, M.; Csizi, K.-S.; Grimmel, S.; Sobez, J.-G.; Steiner, M.; Türtscher, P. L.; Unsleber, J. P.; Reiher, M. qcscine/xtb_wrapper: Release 3.0.2 (3.0.2). 2025; `https://doi.org/10.5281/zenodo.17300808`.

(10) Petrus, E.; Hunkeler, L. A.; Reiher, M.; von Gunten, U.; Hofstetter, T. B. Automated reaction exploration of ozonation processes for model olefins in water. *Environ. Sci. Technol.* **2025**, *59*, https://doi.org/10.1021/acs.est.5c16167.

(11) Steiner, M.; Reiher, M. A human-machine interface for automatic exploration of chemical reaction networks. *Nat. Comm.* **2024**, *15*, 3680, https://doi.org/10.1038/s41467-024-47997-9.

(12) Bannwarth, C.; Caldeweyher, E.; Ehlert, S.; Hansen, A.; Pracht, P.; Seibert, J.; Spicher, S.; Grimme, S. Extended tight-binding quantum chemistry methods. *WIREs Comput Mol Sci* **2021**, *11*, e1493, https://doi.org/10.1002/wcms.1493.

(13) Sobez, J.-G.; Reiher, M. Molassembler: molecular graph construction, modification, and conformer generation for inorganic and organic molecules. *J. Chem. Inf. Model.* **2020**, *60*, 3884–3900, https://doi.org/10.1021/acs.jcim.0c00503.

(14) Bensberg, M.; Grimmel, S.; Sobez, J.-G.; Steiner, M.; Unsleber, J. P.; Reiher, M. qcscine/molassembler: Release 2.0.1. 2023; `https://doi.org/10.5281/zenodo.10159517`.

(15) Kim, Y.; Kim, W. Y. Universal structure conversion method for organic molecules: from atomic connectivity to three-dimensional geometry. *Bull. Korean Chem. Soc.* **2015**, *36*, 1769–1777, https://doi.org/10.1002/bkcs.10334.

(16) Jensen Research Group xyz2mol: Converts an XYZ file to an RDKit mol object. `https://github.com/jensengroup/xyz2mol`, 2022; `https://github.com/jensengroup/xyz2mol`, Accessed: 2026-01-30.

(17) Neumann, S.; Meier, R.; Wenk, M.; Elapavalore, A.; Nishioka, T.; Schulze, T.; Stravs, M.; Tsugawa, H.; Matsuda, F.; Schymanski, E. L. MassBank: an open and FAIR mass spectral data resource. *Nucleic Acids Research* **2026**, *54*, D601–D606, https://doi.org/10.1093/nar/gkaf1193.

(18) MassBank Consortium MassBank Record: MSBNK-EPA-ENTACT_AGILENT002123. `https://massbank.eu/MassBank/RecordDisplay?id=MSBNK-EPA-ENTACT_AGILENT002123`.

(19) Dührkop, K.; Fleischauer, M.; Ludwig, M.; Aksenov, A. A.; Melnik, A. V.; Meusel, M.; Dorrestein, P. C.; Rousu, J.; Böcker, S. SIRIUS 4: A rapid tool for turning tandem mass spectra into metabolite structure information. *Nat. Methods* **2019**, *16*, 299–302, https://doi.org/10.1038/s41592-019-0344-8.

(20) Kamada, T.; Kawai, S. An algorithm for drawing general undirected graphs. *Inf. Process. Lett* **1989**, *31*, 7–15, https://doi.org/10.1016/0020-0190(89)90102-6.

(21) Fruchterman, T. M. J.; Reingold, E. M. Graph drawing by force-directed placement. *Softw. Pract. Exper.* **1991**, *21*, 1129–1164, https://doi.org/10.1002/spe.4380211102.

(22) Hagberg, A. A.; Schult, D. A.; Swart, P. J. Exploring network structure, dynamics, and function using networkX. *Python in Science Conference* **2008**, https://doi.org/10.25080/TCWV9851.

(23) MacQueen, J. B. Some Methods for Classification and Analysis of MultiVariate Observations. Proc. of the fifth Berkeley Symposium on Mathematical Statistics and Probability. 1967; pp 281–297, https://doi.org/10.1002/0471649643.

(24) Rousseeuw, P. J. Silhouettes: A graphical aid to the interpretation and validation of cluster analysis. *J. Comput. Appl. Math* **1987**, *20*, 53–65.

(25) Tarjan, R. Depth-First Search and Linear Graph Algorithms. *SIAM Journal on Computing* **1972**, *1*, 146–160, https://doi.org/10.1137/0201010.
